# Antidiabetic Activity of a Flavonoid-Rich Extract From *Sophora davidii* (Franch.) Skeels in KK-Ay Mice via Activation of AMP-Activated Protein Kinase

**DOI:** 10.3389/fphar.2018.00760

**Published:** 2018-07-16

**Authors:** Yun Huang, Ji Hao, Di Tian, Yanzhang Wen, Ping Zhao, Hao Chen, Yibin Lv, Xinzhou Yang

**Affiliations:** ^1^School of Pharmaceutical Sciences, South-Central University for Nationalities, Wuhan, China; ^2^School of Life Sciences, South-Central University for Nationalities, Wuhan, China; ^3^College of Pharmacy, Guangxi University of Chinese Medicine, Nanning, China

**Keywords:** antidiabetic, *Sophora davidii* (Franch.) Skeels, KK-Ay mice, GLUT4, AMPK

## Abstract

The present study was undertaken to investigate the hypoglycemic activity and potential mechanisms of action of a flavonoid-rich extract from *Sophora davidii* (Franch.) Skeels (SD-FRE) through *in vitro* and *in vivo* studies. Four main flavonoids of SD-FRE namely apigenin, maackiain, leachianone A and leachianone B were purified and identified. *In vitro*, SD-FRE significantly promoted the translocation and expression of glucose transporter 4 (GLUT4) in L6 cells, which was significantly inhibited by Compound C (AMPK inhibitor), but not by Wortmannin (PI3K inhibitor) or Gö6983 (PKC inhibitor). These results indicated that SD-FRE enhanced GLUT4 expression and translocation to the plasma membrane via the AMPK pathway and finally resulted in an increase of glucose uptake. *In vivo*, using a spontaneously type 2 diabetic model, KK-Ay mice received intragastric administration of SD-FRE for 4 weeks. As a consequence, SD-FRE significantly alleviated the hyperglycemia, glucose intolerance, insulin resistance and hyperlipidemia in these mice. Hepatic steatosis, islet hypertrophy and larger adipocyte size were observed in KK-Ay mice. However, these pathological changes were effectively relieved by SD-FRE treatment. SD-FRE promoted GLUT4 expression and activated AMPK phosphorylation in insulin target tissues (muscle, adipose tissue and liver) of KK-Ay mice, thus facilitating glucose utilization to ameliorate insulin resistance. Regulation of ACC phosphorylation and PPARγ were also involved in the antidiabetic effects of SD-FRE. Taken together, these findings indicated that SD-FRE has the potential to alleviate type 2 diabetes.

## Introduction

Diabetes has become one of the most challenging health problems on a global scale. The International Diabetes Federation (IDF) estimated that in 2017 there were 451 million (age 18–99 years) people with diabetes worldwide and this number was predicted to rise to 693 million by 2045 (Cho et al., 2018). Diabetes mellitus is a complex disorder characterized by abnormal metabolism of carbohydrates, lipids and proteins, and type 2 diabetes mellitus (T2DM) is the most prevalent form of diabetes, which accounts for more than 90% of diabetic cases (Nolan et al., 2011). The main pathogenesis of T2DM is complex, including insulin resistance in target tissues (primarily muscle, adipose tissue, and liver), relatively insufficient insulin secretion, and subsequent pancreatic beta cell dysfunction (Nyenwe et al., 2011). There are several groups of antidiabetic drugs available to treat T2DM including sulfonylureas, biguanides, thiazolidinediones, glinides, α-glucosidase inhibitors, etc. Although they have positive effects, some side effects such as hypoglycemia, weight gain, gastrointestinal disturbances and edema are also associated with them (Alam et al., 2016). Therefore, looking for more effective and safer hypoglycemic agents is a growing need.

Glucose transporter 4 (GLUT4), which transports glucose through the cell membrane into the cell is the rate limiting step of glucose uptake in the body (Huang and Czech, 2007). Selective disruption of GLUT4 expression in muscle or adipose tissue leads to global insulin resistance and thereby increase the risk of developing diabetes (Zisman et al., 2000; Abel et al., 2001). As the important protein for preserving whole-body glucose homeostasis, GLUT4 is considered to be a potential therapeutic targets for the treatment of T2DM (Morgan et al., 2011). In recent years, natural products have become a re-emergent trend in clinical medicine and postulated as potential treatment for T2DM due to their efficacy and minimal side effects (Li et al., 2017). To date, several natural products have been reported to exhibit significant antidiabetic activity via targeting GLUT4, including jicama extract (Park et al., 2016), cinnamic acid (Lakshmi et al., 2009), and ergosterol (Xiong et al., 2018).

To look for potential hypoglycemic agents from natural products, a cell-based GLUT4 translocation assay system with L6 cells stably over-expressing IRAP-mOrange was established. Potential antidiabetic extracts, fractions, and individual compounds from natural products samples were screened by evaluating their effects on promoting GLUT4 translocation using confocal microscopy. As a consequence, we have found that a flavonoid-rich extract from roots of *Sophora davidii* (Franch.) Skeels (SD-FRE) showed a promising positive activity on promoting GLUT4 translocation.

*Sophora davidii* (Franch.) Skeels belongs to the leguminous family, growing on hillside, wayside or bushes at an altitude of 2,000–3,500 m. It is mainly distributed in the Guizhou, Yunnan, Sichuan and Ningxia provinces in China (Tai et al., 2011). The flowers, roots, leaves and fruits of *S. davidii* have been traditionally used as folk medicines by local people. The roots of *S. davidii* had the functions of clearing heat, soothing a sore throat, cooling the blood and reducing swelling, and have been used to treat sore throat, hematochezia, cough and dysentery, etc. (Zhao, 2004). To the best of our knowledge, the hypoglycemic potential of *S. davidii* has not been investigated or reported to date. In the present study, we observed that SD-FRE displayed a strong effect in promoting GLUT4 translocation and improving glucose uptake in L6 rat skeletal muscle cells. This observation has led to the hypothesis that SD-FRE will be effective in alleviating T2DM. We tested this hypothesis using L6 cells as well as in KK-Ay mice. Additionally, proteins related to glucose and lipid metabolism were evaluated to demonstrate the molecular mechanisms underlying the effects of SD-FRE on T2DM.

## Materials and Methods

### Instruments

The NMR spectra were recorded on an AVANCE III 600 MHz spectrometer equipped with 1.4 mm heavy wall Micro NMR tubes (NORELL, Landisville, NJ, United States). Liquid chromatography-photo-diode array-electrospray ionization mass spectrometry (LC-PDA-ESIMS) analyses were recorded on a Waters ACQUITY SQD MS system (Waters, Milford, MA, United States) connected to a Waters 1525 high performance liquid chromatography (HPLC) with a 2998 Photodiode Array Detector (Waters, Milford, MA, United States) and a Waters Sunfire C18 column (5 μm, 4.6 × 150 mm) (Waters, Ireland). Semi-preparative HPLC was carried out on a Waters 2535 HPLC fitted with a 2998 Photodiode Array Detector and a 2707 Autosampler (Waters). Separations were performed on a Waters SunfireTM C18 column (5 μm, 10 × 150 mm) (Waters, Ireland). All the solvents used for chromatography were of HPLC grade and all the other chemicals were of analytical-reagent grade. HPLC-grade methanol and acetonitrile were purchased from Merck Chemical Company (Darmstadt, Germany). Sephadex LH-20 gel was obtained from GE Health Care (Uppsala, Sweden).

### Chemicals and Reagents

α-Minimum Essential Medium (α-MEM) and fetal bovine serum (FBS) and antibiotics (100 U/mL penicillin and 100 μg/mL streptomycin) were obtained from Hyclone (Logan, UT, United States). Compound C {6-[4-(2-piperidin-1-ylethoxy)phenyl]-3-pyridin-4-ylpyrazolo[1,5-a] pyrimidine} was purchased from Calbiochem (San Diego, CA, United States). Wortmannin {(1S,6bR,9aS,11R,11bR)-11-(acetyloxy)-1,6b,7,8,9a,10,11,11b-octahydro-1-(methoxy-methyl)-9a,11b-dimethyl-(3H-Furo[4,3,2-de]indeno[4,5-h]-2-benzopyran-3,6,9-trione} was purchased from Selleckchem (Houston, TX, United States). Gö6983 {3-[1-[3-(Dimethylamino)propyl]-5-methoxy-1H-indol-3-yl]-4-(1H-indol-3-yl)-1H-pyrrole-2,5-dione}was purchased from EMD Millipore (Billerica, MA, United States). AICAR (5-aminoimidazole-4-carboxamide1-b-D-ribofuranoside) was purchased from Sigma-Aldrich (Shanghai) Trading Co., Ltd. (Shanghai, China). The 2-[N-(7-nitrobenz-2-oxa-1,3-diaxol-4-yl)amino]-2-deoxyglucose (2-NBDG) assay kit was purchased from Cayman Chemical (Ann Arbor, MI, United States). The insulin Elisa assay kit was obtained Jiancheng Bioengineering Institute (Nanjing, Jiangsu Province, China). Triglycerides (TG), total cholesterol (TC), free fatty acid (FFA) kits were all purchased from Jiancheng Bioengineering Institute, Nanjing, China. BCA protein quantification kit was purchased from Beyotime Biotechnology (Nantong, Jiangsu Province, China). Antibodies of β-Actin, GLUT4, AMPKα, Phospho-AMPKα (Thr172) (p-AMPK), Acetyl-CoA Carboxylase (ACC), Phospho-Acetyl-CoA Carboxylase (Ser79) (p-ACC), PPARγ and the corresponding secondary antibodies were obtained from Cell Signaling Technology (Danvers, MA, United States). Enhanced chemiluminescence (ECL) kits were obtained from Chongqi Biospes Co., Ltd.

### Animals

The care and use of animals and all procedures involving animals were carried out in accordance with the Guidelines for Animal Experiments of SCUN and were approved by the Animal Ethics Committee of SCUN (Approval Number: S08917111E). KK-Ay mice, a widely used animal model of obesity and T2DM, exhibits obesity, hyperglycaemia and hyperinsulinaemia, and is an ideal animal model for studying insulin resistance and T2DM (Srinivasan and Ramarao, 2007). Eight-week old male KK-Ay mice and C57BL/6J mice of Specific Pathogen Free (SPF) grade were purchased from the Beijing HFK Bioscience Co., Ltd. All mice were housed with enough food and water under standard conditions of temperature 23 ± 2°C, 50–60% relative humidity, with a constant 12 h light–dark cycle.

### Plant Material and Preparation of SD-FRE

The roots of *S. davidii* (Franch.) Skeels were collected from Xiuwen county, Guizhou province, China in June 2014. The identification was done by Professor Dingrong Wan of School of Pharmaceutical Sciences, South-Central University for Nationalities (SCUN), Wuhan, China. A voucher specimen (SC0801) is deposited in School of Pharmaceutical Sciences, SCUN, Wuhan, China. Air-dried roots of *S. davidii* (500 g) were smashed and extracted sequentially by maceration at room temperature with 80% ethanol (4 × 10 L, 3 days each). The solvents were evaporated at reduced pressure to yield 113 g of residue. The residue was dismissed to slurry by water (1:10), and the slurry was then extracted with petroleum ether (4 × 2.0 L), ethyl acetate (4 × 2.0 L) and *n*-BuOH (4 × 2.0 L), respectively. The solvents were evaporated at reduced pressure to yield petroleum ether extract (17 g), ethyl acetate extract (25 g), and *n*-BuOH extract (38 g), respectively. The petroleum ether extract (16 g) was subjected to column chromatography of D101 macroporous resin of 400 g (Sinopharm Chemical Reagent Co., Ltd., Shanghai, China) eluting with 10, 30, 50, 70, and 95% aq. Ethanol in a gradient manner. The different aq. ethanol eluates were evaporated to dryness, and were subsequently subjected to GLUT4 translocation assay. The 70% aq. ethanol eluted fraction exhibited the strongest GLUT4 translocation activity as the purified flavonoid-rich extract (SD-FRE, 6 g).

### LC-PDA-ESIMS Profiling of SD-FRE

The LC-PDA-ESIMS profiling of SD-FRE was performed according to the standard operating procedure previously described (Huang et al., 2016). Analysis was carried out on a Waters Sunfire C18 column (5 μm, 4.6 × 150 mm) equipped with a Waters Sunfire C18 cartridge as a precolumn. Water (A) and acetonitrile (B) containing 0.1% formic acid were used as mobile phases. The gradient program was used as follows: 0–20 min, 10–100% B; 20–25 min, 100% B. The analysis was performed at the flow rate of 1.0 mL/min. UV–vis spectra were recorded over a range of 200–500 nm at a spectral acquisition rate of 10 scans per second. The eluent was split at a 1:5 ratio before the mass spectrometer. ESI-MS were recorded in both positive and negative ion modes. Mass range was set from 100 to 1,500 m/z. Data acquisition and processing were achieved with MassLynx 4.0 software (Waters, Milford, MA, United States).

### Chemical Characterization of SD-FRE

The separation of SD-FRE was carried by the procedure as previously described (Zheng et al., 2017). 100 mg of SD-FRE was dissolved in 2.0 mL of the mixture solution (50% DMSO: 50% methanol) and the solution was filtered. Two hundred microliter of SD-PRE was subjected to a semipreparative HPLC with a Waters Sunfire C18 HPLC column (5 μm, 19 × 250 mm i.d.) (acetonitrile in water with 0.1% formic acid from 10 to 100%, 25 min, 100% acetonitrile holding for the next 5 min). A total of 4 peak-based fractions were collected manually and 10 injections were repeated. Peaks 1–4 were filtered through a Sephadex LH-20 column (350 × 10 mm, 65% MeOH: 35% CH_2_Cl_2_ containing 0.1% formic acid) to yield 4 pure compounds, **1** (4.3 mg), **2** (6.7 mg), **3** (41.5 mg), and **4** (11.8 mg). Compounds **1** – **4** were dissolved in MeOH-d_4_ for NMR with the amount range of 4.0–6.0 mg for NMR tests.

### Cell Culture

Skeletal muscles are the major site for glucose utilization and play an important role in maintaining glucose homeostasis (Saltiel and Kahn, 2001). L6 cells were from rat skeletal muscle cells and have been usually used to construct simple glucose uptake assay systems (Hutchinson and Bengtsson, 2005). In the present study, L6 cells were cultured in α-MEM, supplemented with 10% FBS and 1% antibiotics (100 U/mL penicillin and 100 μg/mL streptomycin) at 37°C in 5% CO_2_. Cells were subcultured when the density at about 60%. L6 cells were cultured in α-MEM plus 2% FBS and 1% antibiotics at 37°C in 5% CO_2_ for 7 days to form myotubes and the medium was replaced every 2 days. After differentiation, the cells were used for experiment.

### Plasmid and Cell Line Construction

pIRAP-mOrange cDNAs (presented by Professor Xu Tao, Institute of Biophysics, the Chinese Academy of Sciences) were inserted into the pQCXIP plasmid. The retrovirus was prepared by transfecting pQCXIP-IRAP-mOrange, vesicular stomatitis virus G (VSVG), and PHIT60 (including MuLV structural genes, namely gag and pol) at a ratio of 2: 1: 1 through Lipofectamine 2000 (Invitrogen, Waltham, CA, United States) into Plat E cells. 48 h later, the cultural supernatant was collected and viruses were concentrated by super-centrifugation (at 50,000 g, 30 min). L6 cells were infected with freshly prepared viruses at the exponential growth phase. Polybrene (8 μg/mL) was used to improve the efficiency of virus infection. L6 cells with fluorescence were isolated by fluorescence activated cell sorter (FACS) and seeded into 96-well plates. Finally, the single clone with the highest fluorescence intensity following the treatment with insulin (100 nM) was selected.

### Assay of IRAP Translocation

Insulin-regulated aminopeptidase (IRAP) has been identified as a major protein that co-localizes with GLUT4 in insulin-responsive GLUTs storage vesicles (GSVs) and has been successfully used as a reporter molecule that can indirectly reflect GLUT4 translocation (Lampson et al., 2000). The methodology validation can be found as S1 in Supplementary Material. L6 cells stably expressing IRAP-mOrange (L6 IRAP-mOrange) were cultured in α-MEM supplemented with 10% FBS and 1% antibiotics at 37°C in 5% CO_2_. L6 IRAP-mOrange was seeded onto glass cover slips for overnight, and then starved in serum-free α-MEM for 2 h. Afterward, L6 cells were treated with 30 μg/mL SD-FRE or other agents, 10 μM Compound C [an inhibitor of AMP-activated protein kinase (AMPK)], 100 nM Wortmannin [an inhibitor of phosphatidylinositol 3-kinase (PI3K)], and 10 μM Gö6983 [an inhibitor of protein kinase C (PKC)]. L6 IRAP-mOrange was imaged with a laser-scanning confocal microscope LSM 700 (Carl Zeiss, Jena, Germany) to supervise the dynamics of IRAP-mOrange translocation. The photos were taken after the addition of samples by 555 nm excitation laser every 5 min in 30 min. The fluorescence intensity of IRAP-mOrange at the plasma membrane was measured as previously described (Zhao et al., 2009) and was used to reflect the GLUT4 translocation.

### Assay of Glucose Uptake

Glucose uptake in L6 cells was measured by a cell-based 2-NBDG Glucose Uptake Assay Kit. L6 cells were seeded in a 96-well black plate with the density 1 × 10^4^–5 × 10^4^ cells/well in 100 μL α-MEM. After overnight incubation, cells were treated with AICAR (1 mM), SD-FRE (10, 20, and 30 μg/mL) or normal control (0.1% DMSO) in 100 μL glucose-free α-MEM containing 150 μg/mL 2-NBDG. After 24 h incubation, the glucose uptake of L6 cells was measured by the method described in the glucose uptake assay kit.

### Preparation of Protein in L6 Myotubes

L6 cells (5 × 10^5^ cells) were subcultured into 60 mm dishes and cultured for 7 days to form myotubes in 3 mL of α-MEM with 2% FBS. After incubation, the L6 myotubes were treated with AICAR (1 mM), SD-FRE (10, 20, and 30 μg/mL), Compound C (10 μM), or normal control (0.1% DMSO) for 12 h. The cells were then washed with cold PBS, and then lysed using a RIPA protein extraction kit. The whole cell lysate was centrifuged at 15,000 g for 15 min to remove insoluble protein. The protein concentration of lysates was determined using the BCA protein assay kit.

### Animal Experimental Design

All mice were allowed to acclimatize for 1 week. C57LB/6J mice fed with normal diet (Beijing HFK Bioscience Co., Ltd) were taken as a normal control (NC) group. KK-Ay mice were fed with high-fat diet (HFD; 4.73 kcal/g, 20% kcal from protein, 35% kcal from carbohydrates and 45% kcal from fat; MD12032, Medicience Co., Ltd., Yangzhou, China). After 4 weeks, KK-Ay mice with fasting blood glucose (FBG) level more than 11.1 mmol/L were classified as T2DM, and then these mice were randomly divided into four groups of 8 animals each: diabetic control (DC), low dose SD-FRE treatment (SFL, 60 mg/kg bw), high dose SD-FRE treatment (SFH, 120 mg/kg bw), metformin treatment (MET, 200 mg/kg bw). SD-FRE was suspended in 1% Tween 80 and administered to mice by oral gavage at a dose volume of 0.1 mL/10 g body weight. Mice in NC and DC groups were intragastric administered with the same solvent. Mice in all groups received intragastric administration once daily for 4 consecutive weeks.

During the experimental period, body weight and FBG levels of mice were measured once weekly. An oral glucose tolerance test (OGTT) was carried out in overnight-fasted mice at the end of the experiment. Blood samples was collected from the tip of the tail at 0, 30, 60, 90, and 120 min from all groups after 2.0 g/kg glucose oral administration to measure the blood glucose level using a blood glucose meter (One Touch Ultra, Lifescan Inc., Wayne, IL, United States). The area under the curves (AUC) generated from the data collected during the OGTT was calculated. At the end of experiment, all mice were anesthetized and blood was collected. The serum was separated by centrifuging the blood samples at 3,000 g for 15 min. The serum biochemical indexes including TC, TG, low density lipoprotein cholesterol (LDL-C), high density lipoprotein cholesterol (HDL-C) and FFA were evaluated by an automatic biochemical analyzer (Hitachi 7180+ISE, Tokyo, Japan). Serum insulin content was measured by using a rodent insulin ELISA kit. Hepatic TC, TG, and FFA were determined by corresponding assay kits. Homeostasis model assessment of insulin resistance (HOMA-IR), an index of insulin resistance, was calculated according to the following equation: (HOMA-IR index = FBG [mmol/L] × FINS [mIU/L]/22.5), (Bonora et al., 2000).

### Histology and Immunohistochemistry

Part of liver, white adipose tissue (WAT) and pancreas were fixed in 4% formaldehyde and embedded in paraffin for hematoxylin–eosin (HE) staining. Frozen liver tissues embedded in OCT were used for Oil Red O (OR) staining. Parts of pancreas sections were incubated with mouse monoclonal anti-insulin and rabbit anti-glucagons [Sigma-Aldrich (Shanghai) Trading Co., Ltd., Shanghai, China]. They were then incubated with corresponding fluorescent-labeled secondary antibody, Alexa Fluor 594 goat anti-mouse IgG, Alexa Fluor 488 goat anti-rabbit IgG (Invitrogen, Carlsbad, CA, United States). Nuclei were stained with DAPI. Stained sections were photographed with a Nikon Eclipse Ti-SR microscope equipped with a Nikon DS-U3 digital camera (Nikon Incorporation, Tokyo, Japan)

### Western Blot Analysis in L6 Cells and Insulin Target Tissues

Parts of frozen muscle, liver, and WAT were thawed, weighed, roughly cut, and homogenized with the RIPA protein extraction kit. The mixture was lysed for 30 min on ice, and centrifuged at 12,000 g for 15 min at 4°C, whereby non soluble material was discarded, and the protein concentration was measured as described above. Collected proteins in cells and tissues were subjected to western blot analysis according to the methods described previously (Huang et al., 2016; Yang et al., 2017). Briefly, an equivalent amount of samples were subjected to 8 or 10% sodium dodecyl sulfate (SDS)-polyacrylamide gel electrophoresis and transferred to a polyvinylidene difluoride membrane (Pall Corporation, Washington, United States), and the membranes were incubated with primary antibodies and HRP-conjugated secondary antibodies. Then protein bands were incubated in ECL kits, and detected and quantified by a ChemiDoc XRS (Bio-Rad, Hercules, CA, United States).

### Statistical Analysis

Data were shown as mean ± standard error of the mean (SEM). Differences between groups were analyzed with one-way analysis of variance (ANOVA), followed by Tukey’s *post hoc* test using Graphpad prime software. A probability (P) value of less than 0.05 was considered statistically significant.

## Results

### Chemical Characterization of SD-FRE

**FIGURE 1:**
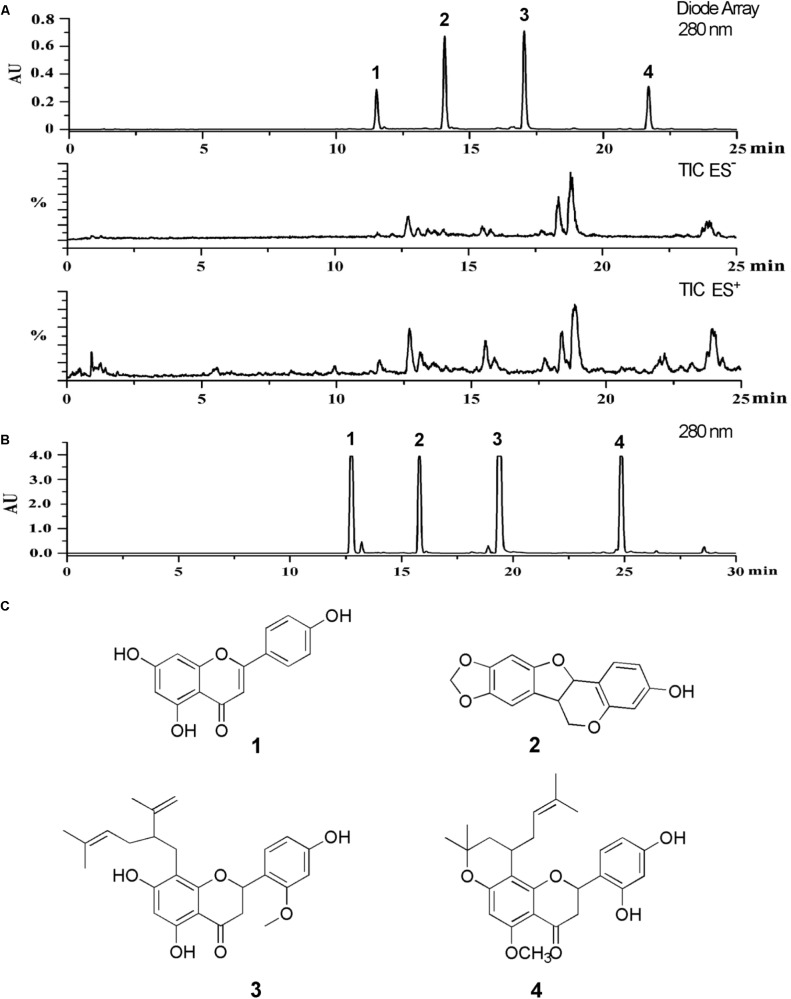
**(A)** The LC-PDA-ESIMS analysis of SD-FRE is shown at 280 nm with peak labeling corresponding to compounds 1–4. **(B)** The optimized semi-preparative HPLC separation of the SD-FRE (10 mg in 200 μL DMSO) is shown at 280 nm with peaks 1–4 collected for microprobe NMR and further purified if required. **(C)** Structures of 4 flavonoids from SD-FRE.

The LC-PDA-ESIMS profiling of SD-FRE was shown in **Figure 1A**. To achieve good resolution of critical HPLC peaks, the large scale preparative conditions were optimized with an optimized separation of holding for 30 min to purify target compounds. A typical semi-preparative HPLC chromatogram obtained with an injection of 10 mg of extract is shown in **Figure 1B**. A total of four peaks were collected and submitted to further purification with Sephadex LH-20 column to afford four pure compounds for further NMR and MS spectroscopic analysis. NMR spectra were recorded with 1.4 mm heavy wall Micro NMR tubes. The purity was tested by analytical HPLC under same conditions as shown in **Figure 1A**. The four major peaks were determined as apigenin (**1**) (Itokawa et al., 1981), maackiain (**2**) (Peng et al., 2016), leachianone A (**3**) (Iinum et al., 1990) and leachianone B (**4**) (Matsuo et al., 2003) by comparison of their MS, UV, and NMR spectra along with published reference data (**Figure 1C**). Spectroscopic data of compounds 1–4 could be found in S3–S14 of Supplementary Material.

### SD-FRE Increased Glucose Uptake, Enhanced GLUT4 Expression and Translocation Through AMPK Pathway

**FIGURE 2:**
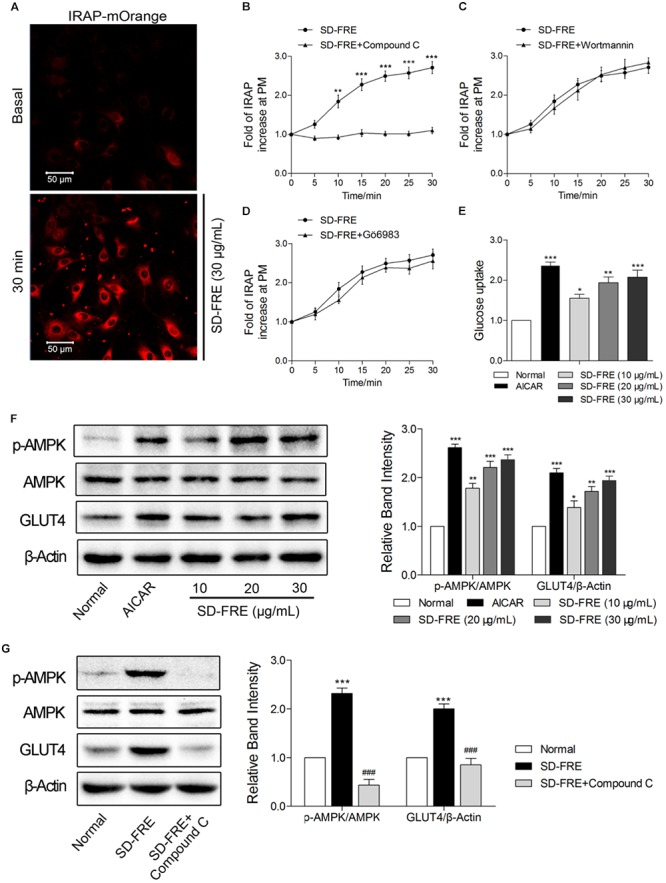
Effects of SD-FRE *in vitro*. **(A)** IRAP fluorescence in L6 IRAP-mOrange was measured by confocal microscope. Following the addition of SD-FRE (30 μg/mL), IRAP fluorescence intensity at the plasma membrane was increased significantly at 30 min. Scale bar, 50 μm. **(B–D)** Time course of the change in fluorescence induced by SD-FRE with inhibitors (Compound C, Wortmannin, Gö6983) between 0 and 30 min. ^∗∗^*P* < 0.01, ^∗∗∗^*P* < 0.001 compared with SD-FRE + Compound C. **(E)** Glucose uptake was measured by 2-NBDG assay. AICAR and SD-FRE (10, 20, and 30 μg/mL) increased glucose uptake, respectively. ^∗^*P* < 0.05, ^∗∗^*P* < 0.01, ^∗∗∗^*P* < 0.001 compared with control. **(F)** AICAR and SD-FRE (10, 20, and 30 μg/mL) induced increases in AMPK phosphorylation and GLUT4 expression in L6 cells. ^∗^*P* < 0.05, ^∗∗^*P* < 0.01, ^∗∗∗^*P* < 0.001 compared with normal. **(G)** Compound C inhibited the SD-FRE (30 μg/mL) induced AMPK phosphorylation and GLUT4 expression. Data are mean ± SEM, shown as relative band intensity compared with normal control (*n* = 3). ^∗∗∗^*P* < 0.001 compared with normal, ^###^*P* < 0.001 compared with SD-FRE group.

The effect of SD-FRE on GLUT4 translocation in L6 cells which stably expressed IRAP-mOrange was tested. Due to the strong colocalization between IRAP and GLUT4, the trafficking of IRAP-mOrange in L6 cells were specifically examined using confocal microscopy to reflect the GLUT4 translocation. Following the addition of SD-FRE, a significant increase in IRAP-mOrange fluorescence was observed at the plasma membrane (**Figure 2A**), indicating that SD-FRE significantly promoted GLUT4 translocation to the plasma membrane in L6 cells. Previous studies have reported that AMPK, PI3K/Akt and PKC pathways were involved in GLUT4 translocation and expression (Thong et al., 2005; Tsuchiya et al., 2013; Ramachandran and Saravanan, 2015). Thus, corresponding pathway inhibitors were added to further investigated the mechanism underlying SD-FRE-induced GLUT4 translocation, and we found that the significant increase of IRAP fluorescence intensity at the plasma membrane induced by SD-FRE was completely inhibited by the addition of AMPK inhibitor Compound C (**Figure 2B**). However, the addition of either the PI3K inhibitor Wortmannin (**Figure 2C**) or the PKC inhibitor Gö6983 (**Figure 2D**) had no significant effects on the IRAP trafficking response. These results implied that SD-FRE promoted GLUT4 translocation by specifically activating AMPK, but did not involve PI3K and PKC. At the same time, the addition of SD-FRE increased glucose uptake significantly in L6 cells (**Figure 2E**). In the later study, western blotting analysis was conducted to confirm this assumption. We found that treating L6 cells with SD-FRE significantly increased AMPK phosphorylation level compared with the normal control, corresponding to increased phosphorylation of AMPK, the expression of GLUT4 was also significantly enhanced (**Figure 2F**). However, when adding SD-FRE accompanied with Compound C, SD-FRE-induced AMPK phosphorylation and GLUT4 expression were totally repressed (**Figure 2G**). Taken together, these results suggested that SD-FRE increased glucose uptake in L6 cells through activating AMPK and promoting GLUT4 expression and translocation.

### SD-FRE Improved Glucose Metabolism in KK-Ay Mice

**FIGURE 3:**
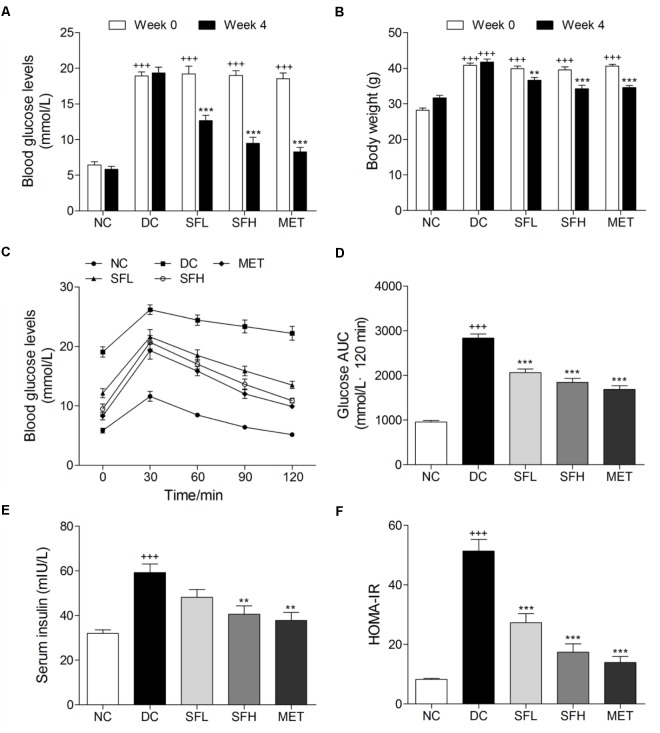
SD-FRE improved glucose metabolism in KK-Ay mice. FBG levels **(A)** and body weight **(B)** of KK-Ay mice at 0 and 4 weeks of SD-FRE treatment. Blood glucose levels **(C)** and AUC of glucose **(D)** during OGTT. Serum insulin level **(E)** and values of HOMA-IR in each group **(F)**. Data are mean ± SEM (*n* = 8). ^+++^*p* < 0.001 vs. NC group, ^∗∗^*p* < 0.01, ^∗∗∗^*p* < 0.001 vs. DC group.

After 4 weeks HFD feeding, KK-Ay mice showed significantly greater FBG levels and body weights than normal control mice. During the experimental period, KK-Ay mice in the DC group showed persistent hyperglycemia, combined with obesity. However, these symptoms were gradually ameliorated by SD-FRE treatment during 4 weeks (Supplementary Figure S2). At the end of 4 weeks treatment, FBG levels of mice in SFL, SFH, and MET groups were significantly reduced compared with mice in the DC group, whereas normal control mice in the NC group maintained stable blood glucose levels (**Figure 3A**). Oral administration of SD-FRE for 4 weeks produced significant attenuation in body weight of mice compared to that of the DC group (**Figure 3B**). There were no significant differences in food intake between the DC group and the SD-FRE treated groups during the 4 weeks treatment period (Supplementary Figure S2C). At the end of the experimental period, an OGTT test was performed to determine the whole-body insulin sensitivity. As a result, KK-Ay mice showed impaired glucose tolerance, with a sharp increase in blood glucose levels after oral administration of 2.0 g/kg glucose, which remained at a high level over the next 120 min. In contrast, the rise in blood glucose levels in SFL, SFH, and MET groups were greatly suppressed, and the elevated blood glucose levels were reduced quickly (**Figure 3C**). In addition, AUC values of glucose response over the 120 min period were calculated and found that AUC values constructed from blood glucose levels in treated groups were significantly decreased in comparison with that of the DC group (**Figure 3D**). KK-Ay mice exhibited much higher insulin levels than normal control mice. After 4 weeks of treatment with SD-FRE, serum insulin levels were clearly reduced compared with those in the DC group (**Figure 3E**). And HOMA-IR index in SD-FRE treated groups were significantly decreased compared to the DC group (**Figure 3F**).

### SD-FRE Ameliorated Serum Lipid Levels in KK-Ay Mice

**FIGURE 4:**
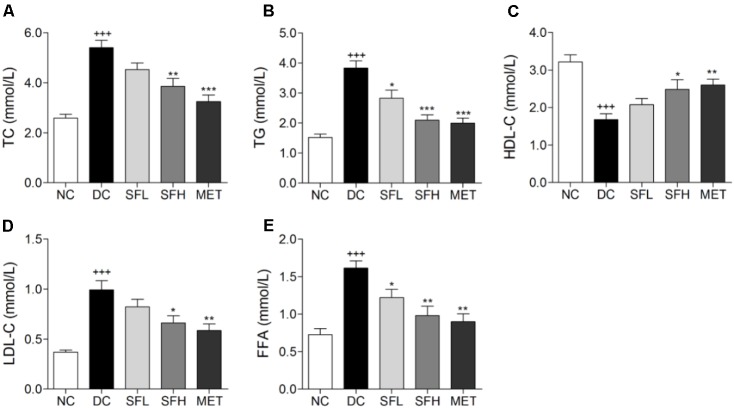
Serum lipid levels were significantly improved by SD-FRE treatment. **(A–E)** Effects of SD-FRE on TC, TG, HDL-C, LDL-C, and FFA levels in the serum. Data are mean ± SEM (*n* = 8). ^+++^*p* < 0.001 vs. NC group, ^∗^*p* < 0.05, ^∗∗^*p* < 0.01, ^∗∗∗^*p* < 0.001 vs. DC group.

Significant lipid metabolism disorders were observed in KK-Ay mice, including a marked increase of TC, TG, FFA, and LDL-C, and decline of HDL-C in serum as compared to normal mice. Following 4 weeks of treatment with SD-FRE, serum TC, TG, FFA, and LDL-C levels were significantly decreased and HDL-C level was markedly increased compared with those in the DC group (**Figure 4**). These results suggested that SD-FRE possessed capability to normalize lipid metabolism.

### SD-FRE Enhanced GLUT4 Expression, Activated AMPK Phosphorylation in Insulin Target Tissues of KK-Ay Mice

**FIGURE 5:**
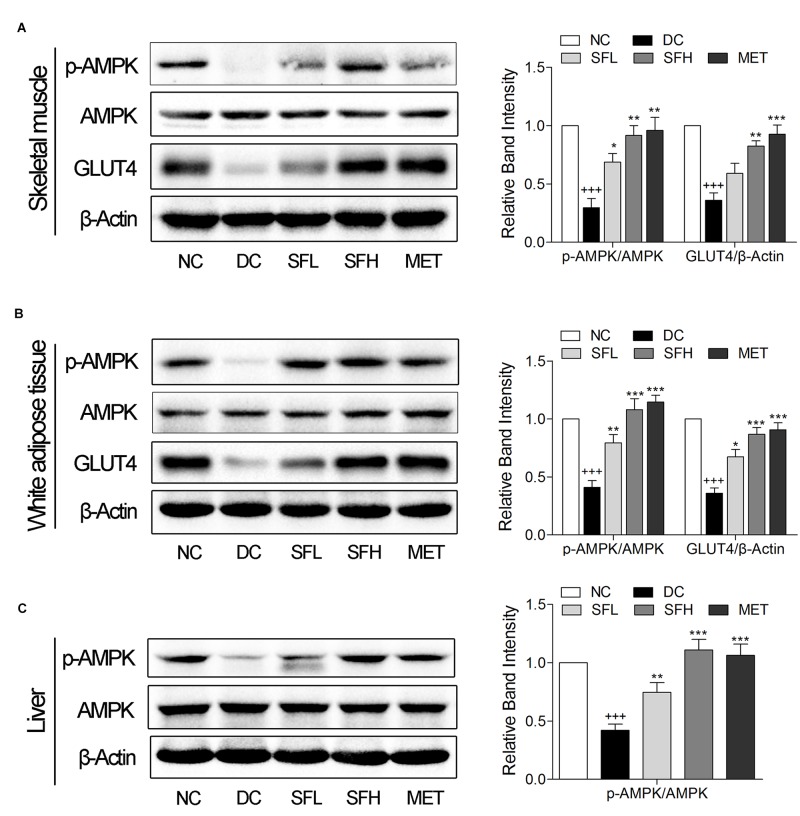
SD-FRE enhanced GLUT4 expression, activated AMPK phosphorylation in insulin target tissues. Western blot analysis of AMPK phosphorylation, GLUT4 expression in skeletal muscle **(A)** and WAT **(B)** of KK-Ay mice. **(C)** Western blot analysis of AMPK phosphorylation in liver of KK-Ay mice. Data are mean ± SEM, shown as relative band intensity compared with NC group (*n* = 3). ^+++^*p* < 0.01 vs. NC group, ^∗^*p* < 0.05, ^∗∗^*p* < 0.01, ^∗∗∗^*p* < 0.001 vs. DC group.

SD-FRE resulted in possible effects on stimulating GLUT4 translocation and expression via AMPK pathway in L6 cells (**Figure 2**). Thus we further examined *in vivo* expression of GLUT4 and p-AMPK in insulin target tissues. The results showed that the expression level of GLUT4 and the ratio of p-AMPK/AMPK in skeletal muscle and WAT of KK-Ay mice in the DC group were lower than that of normal control mice in the NC group. Treatment with SD-FRE and metformin significantly increased GLUT4 expression and AMPK phosphorylation in skeletal muscle and WAT of KK-Ay mice in SFL, SFH, and MET groups compared with mice in the DC group (**Figures 5A,B**). Moreover, SD-FRE treatment reversed the reduced phosphorylation of AMPK in the livers of KK-Ay mice (**Figure 5C**).

### SD-FRE Ameliorated Hepatic Steatosis in KK-Ay Mice

**FIGURE 6:**
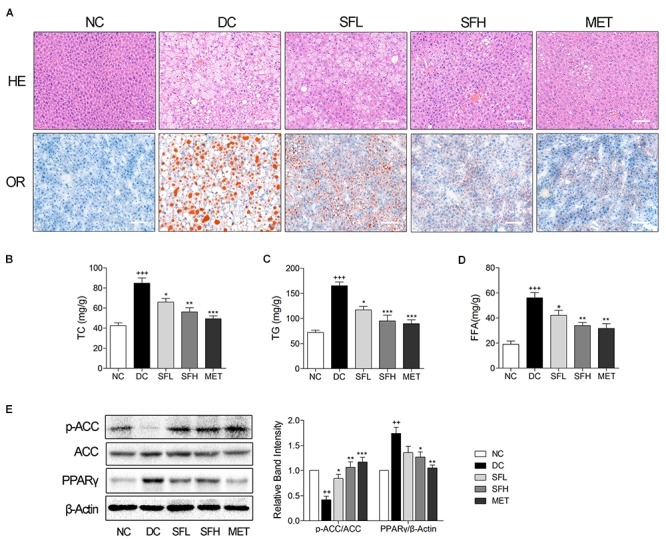
SD-FRE treatment ameliorated hepatic steatosis in KK-Ay mice. **(A)** HE and OR staining of the liver from KK-Ay mice. Scale bar, 50 μm. **(B–D)** The contents of TC, TG, and FFA in the liver from KK-Ay mice. **(E)** Western blot analysis of ACC phosphorylation and PPARγ expression in liver. Data are mean ± SEM, shown as relative band intensity compared with NC group (*n* = 3). ^++^*P* < 0.01, ^+++^*P* < 0.001 vs. NC group, ^∗^*P* < 0.05, ^∗∗^*P* < 0.01, ^∗∗∗^*P* < 0.001 vs. DC group.

Hepatic steatosis and empty lipid vacuoles were observed in the livers of KK-Ay mice in the DC group, whereas livers of control mice in the NC group appeared normal. In contrast, treatment with SD-FRE ameliorated hepatic steatosis, and lowered the hepatic lipid droplet accumulation compared to the DC group (**Figure 6A**, panel HE). We also examined hepatic lipid by Oil red O staining, and found that SD-FRE treatment suppressed accumulation of lipid (**Figure 6A**, panel OR). Additionally, higher hepatic TG, TC and FFA contents were observed in KK-Ay mice in the DC group, nevertheless, both these parameters were significantly decreased following SD-FRE treatment (**Figures 6B–D**). To gain some insights into the molecular mechanism for these positive effects, we further assessed ACC and PPARγ. ACC, an important downstream target of AMPK and a multi-domain enzyme for fatty acid synthesis and conversion of acetyl-CoA tomalonyl-CoA, plays an important role in lipid biosynthesis (Ruderman et al., 1999; Assifi et al., 2005). PPARγ is a master regulator of adipogenesis, and plays an essential role in lipid and glucose metabolism (Lehrke and Lazar, 2005). Previous studies have reported that hepatic steatosis is associated with elevated PPARγ expression in models of diabetes or obesity (Memon et al., 2000; Bedoucha et al., 2001). As a consequence, the ACC phosphorylation was reduced in the DC group compared to the NC group, which is consistent with the increased liver TG, TC and FFA levels. Consistent with the upstream activation of AMPK discussed above, treatment with SD-FRE also enhanced the phosphorylation of ACC in the liver of KK-Ay mice (**Figure 6E**). As expected, KK-Ay mice in the DC group showed markedly increased expression of PPARγ in the liver compared to control mice in the NC group. Following 4 weeks of treatment with SD-FRE, PPARγ levels in the liver of KK-Ay mice were significantly decreased (**Figure 6E**).

### SD-FRE Reduced Adipocytes Size in WAT of KK-Ay Mice

**FIGURE 7:**
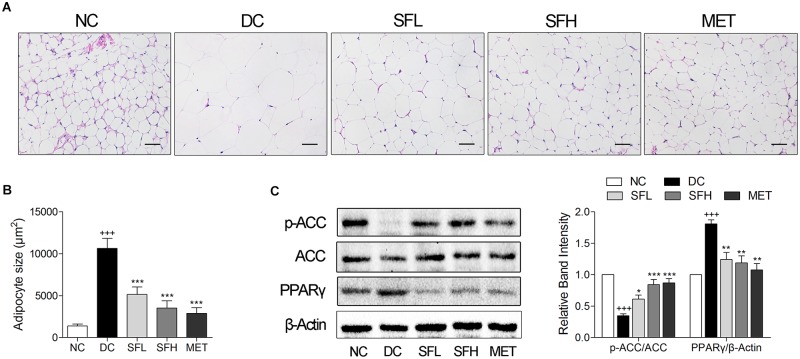
SD-FRE treatment reduced adipocytes size in WAT of KK-Ay mice. **(A)** HE staining of WAT from KK-Ay mice. Scale bar, 50 μm. **(B)** Quantified average adipocyte size in WAT. **(C)** Western blot analysis of ACC phosphorylation and PPARγ expression in WAT. Data are mean ± SEM, shown as relative band intensity compared with NC group (*n* = 3). ^+++^*P* < 0.001 vs. NC group, ^∗^*P* < 0.05, ^∗∗^*P* < 0.01, ^∗∗∗^*P* < 0.001 vs. DC group.

Histological analysis of the WAT and quantification of adipocytes size revealed that larger adipocyte size in WAT was observed in KK-Ay mice of the DC group in correlation to higher body weight. After 4 weeks of treatment with SD-FRE and metformin, mean values of adipocytes size were significantly smaller in SFL, SFH, and MET groups compared with the DC group (**Figures 7A,B**). Furthermore, expression of PPARγ and the phosphorylation of ACC in WAT were also detected and it was found that PPARγ level was higher in WAT of KK-Ay mice in the DC group than that of mice in the NC group. Following 4 weeks of treatment, SD-FRE significantly decreased the expression of PPARγ. The ratio of p-ACC/ACC was significantly decreased in the DC group compared with the NC group, whereas SD-FRE treatment significantly up-regulated the phosphorylation of ACC in the liver (**Figure 7C**), in accordance with increased phosphorylation of AMPK discussed above (**Figure 5C**).

### SD-FRE Ameliorated Pancreatic Islet Hypertrophy in KK-Ay Mice

**FIGURE 8:**
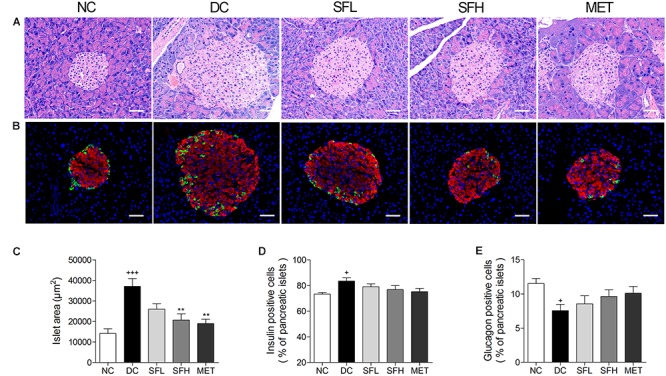
SD-FRE treatment reversed islet hypertrophy in pancreas of KK-Ay mice. **(A)** Pancreas sections were stained with HE and observed under light microscopy. **(B)** Pancreas sections were stained with insulin (red), glucagon (green) and DAPI (blue), observed under fluorescence microscopy. Scale bar, 50 μm. Quantified average islet area **(C)**, the percentage of insulin-positive beta cells **(D)** and glucagon-positive alpha cells **(E)** in pancreatic islets. Data are mean ± SEM. *n* = 4 sections per group. ^+^*p* < 0.05, ^+++^*p* < 0.001 vs. NC group, ^∗∗^*p* < 0.01 vs. DC group.

HE staining of pancreatic islets in the NC group showed a normal islet morphology. Accompanied with hyperinsulinemia, islet cell hypertrophy and increased islet area were observed in the pancreas of KK-Ay mice in the DC group, an overt sign of insulin resistance, which is consistent with previous reports (Moroki et al., 2013). Treatment with SD-FRE and metformin effectively reversed the islet hypertrophy in pancreas of KK-Ay mice (**Figures 8A,C**). Immunofluorescence staining showed that the percentage of insulin-positive beta cells was significantly increased and the percentage of glucagon-positive alpha cells was decreased in islets of KK-Ay mice compared with normal mice, and these changes were ameliorated in the treatment groups (**Figures 8B,D,E**).

## Discussion

Based on our established GLUT4 translocation assay system, we found that SD-FRE displayed a strong effect in promoting GLUT4 translocation and stimulating glucose uptake in L6 cells, indicating that SD-FRE possessed potential hypoglycemic effects. Then, further *in vitro* and *in vivo* studies were conducted to evaluate the potential activity of SD-FRE on T2DM.

GLUT4 is a glucose transporter expressed primarily in adipose and muscle tissues, and is a key regulator of whole-body glucose homeostasis (Leto and Saltiel, 2012). A rapid translocation of GLUT4 to the plasma membrane could result in an increase of glucose uptake (Huang and Czech, 2007). We found that SD-FRE significantly stimulated GLUT4 translocation and increased glucose uptake of L6 cells (**Figures 2A,E**). Previous studies have reported that AMPK, PI3K/Akt, and PKC signaling pathways are involved in GLUT4 translocation and expressing (Thong et al., 2005; Tsuchiya et al., 2013; Ramachandran and Saravanan, 2015). Thus, inhibitors of these signaling pathways were used to detect whether these signaling pathways were involved in SD-FRE-induced stimulation of GLUT4 translocation. We found that SD-FRE-induced GLUT4 translocation was completely inhibited by the known AMPK inhibitor, Compound C (**Figure 2B**), suggesting that SD-FRE stimulated GLUT4 translocation via the AMPK pathway. Further, western blotting analysis showed that SD-FRE promoted AMPK phosphorylation and GLUT4 expression in L6 cells (**Figure 2F**). These *in vitro* findings indicated that SD-FRE via AMPK pathway enhanced GLUT4 expression and translocation to the plasma membrane, finally resulting in an increase of glucose uptake in L6 cells.

To investigate possible effects and potential mechanisms of SD-FRE on T2DM *in vivo*, SD-FRE was administered to KK-Ay mice for 4 weeks. KK-Ay mice, a widely used animal model of obesity and T2DM, exhibits metabolic characteristics that are highly comparable to human T2DM, such as hyperglycemia, obesity, hyperinsulinaemia, dyslipidemia (Srinivasan and Ramarao, 2007). Hyperinsulinemia is a characteristic of insulin resistance and a precursor to T2DM. OGTT and HOMA-IR are simple and widely accepted methods for indirect assessment of peripheral insulin action *in vivo*. Based on our results, SD-FRE treatment significantly reduced FBG levels, improved oral glucose tolerance, reduced serum insulin level and HOMA value (**Figure 3**). These results suggested that SD-FRE is beneficial in enhancing insulin sensitivity in KK-Ay mice.

Lipid metabolism disorders including obesity, dyslipidemia, and non-alcoholic fatty liver disease always company T2DM (Karimi et al., 2015). Obesity is closely associated with an increased risk of developing insulin resistance and constitutes the leading factor contributing to the development of T2DM (Kahn et al., 2006). The majority of patients with T2DM are overweight or obese. Relieving obesity or limiting body weight gain may be a suitable strategy for T2DM treatment. In the present study, SD-FRE treatment significantly reduced body weight of KK-Ay mice, accompanied by lower food intake (**Figure 3B**). Moreover, blood lipid indices (TC, TG, HDL-C, LDL-C, and FFA) showed improvements following SD-FRE treatment (**Figure 4**). The liver plays an important role in glucose and lipid homeostasis. TG and TC deposition in the liver increased the risk of insulin resistance and also contributed to the pathogenesis of fatty liver (Adiels et al., 2008). Furthermore, SD-FRE treatment significantly reduced the hepatic contents of TG and TC. In addition, histopathological observation of the liver revealed that SD-FRE reduced hypertrophy of hepatocytes and suppressed lipid accumulation (**Figure 6**). These results indicated that SD-FRE has an activity in the control of lipid metabolism disorders in KK-Ay mice.

AMPK is a widely expressed serine kinase, represents an energy sensor and metabolic regulator that regulates energy homeostasis and metabolic stresses by controlling several homeostatic mechanisms, and is acknowledged as a potential therapeutic target in the prevention and treatment of T2DM (Zhang et al., 2009). Activation of AMPK increases GLUT4 expression or translocation to the plasma membrane in an insulin-independent manner, eventually stimulating glucose uptake in peripheral tissues (Huang and Czech, 2007; Leto and Saltiel, 2012). *In vivo*, we found that SD-FRE increased GLUT4 expression and AMPK phosphorylation in insulin target tissues (skeletal muscle, WAT, and liver) (**Figure 5**), which was contributes to ameliorating the insulin resistance states of the whole body, reducing the insulin demand and relieving the burden on beta cells (Shen et al., 2012). Moreover, histopathological observation of the pancreas showed SD-FRE effectively reversed islet hypertrophy and decreased beta cell areas (**Figure 8**). From these results can be concluded that SD-FRE improved insulin sensitivity in KK-Ay mice by activating AMPK and GLUT4.

AMPK also plays an important role in lipid metabolism regulation via action on downstream targets (Hardie, 2003). ACC is an important downstream target of AMPK, a key regulatory enzyme in *de novo* fatty acid biosynthesis and lipogenesis (Ruderman et al., 1999). ACC is inactivated by phosphorylation at Ser 79 upon AMPK activation, which leads to reduced malonyl-CoA content and subsequently decreased fatty acid synthesis and increased fatty acid oxidation, thus reducing excessive storage of triglycerides (Assifi et al., 2005). Consistent with the upstream activation of AMPK above, SD-FRE treatment also enhanced the phosphorylation of ACC in liver and WAT, suppressing its activity, further contributing to reduced synthesis of fatty acids and lipid. Consistently, the concentrations of FFA, TG, and TC in liver were also reduced by SD-FRE treatment (**Figures 6B–D**), resulting in improved hepatic lipid metabolism.

PPARγ, a member of the nuclear receptor superfamily of ligand dependent transcription factors, is dominantly expressed in adipose tissue and plays an essential role in lipid and glucose metabolism (Lehrke and Lazar, 2005). Studies have reported that elevated PPARγ expression level were observed in the liver of mice models of diabetes or obesity, such as in KK-Ay mice (Bedoucha et al., 2001), ob/ob mice and db/db mice (Memon et al., 2000), all of which developed severe hepatic steatosis. Targeted deletion of PPARγ in hepatocytes protected mice against a high-fat diet induced hepatic steatosis (Morán-Salvador et al., 2011). Conversely, a high level of PPARγ in mouse liver resulted in exacerbated hepatic steatosis through activating lipogenic genes, thereby increasing *de novo* lipogenesis and hepatic triglyceride content (Yu et al., 2003). These findings implied that there is a strong relationship between hepatic steatosis and elevated PPARγ expression. In the present study, KK-Ay mice developed obvious hepatic steatosis, accompanied with elevated expression of PPARγ in the liver, which were significantly reversed by SD-FRE treatment (**Figure 6**). Moreover, PPARγ is activated under conditions of adipocyte differentiation (Lehrke and Lazar, 2005). Previous report has indicated that the suppression of adipogenesis is associated with the PPARγ signaling pathway (Kudo et al., 2004). The inhibition of PPARγ expression can successfully induce antiobesity effects. In the present study, decreased expression of PPARγ was observed in KK-Ay mice treated with SD-FRE, accompanied with reduced adipocyte size (**Figure 7**). These observations implicated that inhibiting the expression of PPARγ was involved in the antiobesity effect of SD-FRE.

## Conclusion

*In vitro*, SD-FRE promoted GLUT4 translocation and expression, and increased glucose uptake via the AMPK pathway in L6 cells. *In vivo*, SD-FRE significantly alleviated the hyperglycemia, glucose intolerance, insulin resistance, hyperlipidemia, hepatic steatosis, adipocyte hypertrophy and islet hypertrophy in KK-Ay mice. SD-FRE promoted GLUT4 expression and activated AMPK phosphorylation in insulin target tissues of KK-Ay mice, contributing to ameliorate insulin resistance in T2DM. At the same time, inhibiting the activity of ACC and the expression of PPARγ were also observed, which are implicated in the antidiabetic effects of SD-FRE. Taken together, these findings supported the hypothesis that SD-FRE has the potential to become an effective agent in the management of T2DM. Besides, four flavonoids were obtained from SD-FRE and identified. Flavonoids are widely recognized for their wide range of biological activities, and many of them have been reported to be beneficial in treating diabetes and its complications (Chen et al., 2015). Among these four flavonoids, apigenin has been reported to be effective in improving diabetic conditions. Apigenin has shown an antihyperglycemic effect and a protective effect on pancreatic β-cell destruction in streptozotocin-induced diabetes (Esmaeili and Sadeghi, 2009; Rauter et al., 2010). It can ameliorate dyslipidemia, hepatic steatosis and insulin resistance in high-fat diet-induced obese mice (Jung et al., 2016). Leachianone A, which has exhibited inhibitory activity against sodium-dependent glucose cotransporter 2 (SGLT2) (Yang et al., 2014). The presence of these flavonoids may be responsible for the antidiabetic activity of SD-FRE that we demonstrated in this study.

## Author Contributions

XY contributed to the conception of the study. YH, JH, DT, YW, HC, and YL conducted the experiments and analyzed the data. YH, JH, DT, PZ, and XY wrote the manuscript.

## Conflict of Interest Statement

The authors declare that the research was conducted in the absence of any commercial or financial relationships that could be construed as a potential conflict of interest. The reviewer SC and handling Editor declared their shared affiliation at time of review.

## References

[B1] Abel E. D. Peroni O. Kim J. K. Kim Y. B. Boss O. Hadro E. (2001). Adipose-selective targeting of the glut4 gene impairs insulin action in muscle and liver. *Nature* 409 729–733. 10.1038/35055575 11217863

[B2] Adiels M. Taskinen M. R. Borén J. (2008). Fatty liver, insulin resistance, and dyslipidemia. *Curr. Diab. Rep.* 8 60–64. 10.1007/s11892-008-0011-4 18367000

[B3] Alam F. Islam M. A. Kamal M. A. Gan S. H. (2016). Updates on managing type 2 diabetes mellitus with natural products: towards antidiabetic drug development. *Curr. Med. Chem.* 23 1–37. 10.2174/0929867323666160813222436 27528060

[B4] Assifi M. M. Suchankova G. Constant S. Prentki M. Saha A. K. Ruderman N. B. (2005). AMP-activated protein kinase and coordination of hepatic fatty acid metabolism of starved/carbohydrate-refed rats. *Am. J. Physiol. Endocrinol. Metab.* 289 E794–E800. 10.1152/ajpendo.00144.2005 15956049

[B5] Bedoucha M. Atzpodien E. Boelsterli U. A. (2001). Diabetic KKAy mice exhibit increased hepatic PPAR 1 gene expression and develop hepatic steatosis upon chronic treatment with antidiabetic thiazolidinediones. *J. Hepatol.* 35 17–23. 10.1016/S0168-8278(01)00066-6 11495036

[B6] Bonora E. Targher G. Alberiche M. Bonadonna R. C. Saggiani F. Zenere M. B. (2000). Homeostasis model assessment closely mirrors the glucose clamp technique in the assessment of insulin sensitivity studies in subjects with various degrees of glucose tolerance. *Diabetes Care* 23 57–63. 10857969 10.2337/diacare.23.1.57

[B7] Chen J. Mangelinckx S. Adams A. Wang Z. T. Li W. L. De Impel N. (2015). Natural flavonoids as potential herbal medication for the treatment of diabetes mellitus and its complications. *Nat. Prod. Commun.* 10 187–200. 25920244

[B8] Cho N. H. Shaw J. E. Karuranga S. Huang Y. da Rocha Fernandes J. D. Ohlrogge A. W. (2018). IDF diabetes atlas: global estimates of diabetes prevalence for 2017 and projections for 2045. *Diabetes Res. Clin. Pract.* 138 271–281. 10.1016/j.diabres.2018.02.023 29496507

[B9] Esmaeili M. A. Sadeghi H. (2009). Pancreatic β-cell protective effect of rutin and apigenin isolated from *Teucrium polium*. *Pharmacologyonline* 2 341–353.

[B10] Hardie D. G. (2003). Minireview: the AMP-activated protein kinase cascade: the key sensor of cellular energy status. *Endocrinology* 144 5179–5183. 10.1210/en.2003-0982 12960015

[B11] Huang M. Deng S. H. Han Q. Q. Zhao P. Zhou Q. Zheng S. J. (2016). Hypoglycemic activity and the potential mechanism of the flavonoid rich extract from *Sophora tonkinensis* gagnep. in KK-Ay mice. *Front. Pharmacol.* 7:288. 10.3389/fphar.2016.00288 27656144 PMC5011294

[B12] Huang S. H. Czech M. P. (2007). The GLUT4 glucose transporter. *Cell Metab.* 5 237–252. 10.1016/j.cmet.2007.03.006 17403369

[B13] Hutchinson D. S. Bengtsson T. (2005). α1A-adrenoceptors activate glucose uptake in L6 muscle cells through a phospholipase C-, phosphatidylinositol-3 kinase-, and atypical protein kinase C-dependent pathway. *Endocrinology* 146 901–912. 10.1210/en.2004-1083 15550506

[B14] Iinum M. Tanak T. Mizuno M. Shirataki Y. Yokoe I. Komatsua M. (1990). Two flavanones in *Sophora leachiano* and some related structures. *Phytochemistry* 29 2667–2669. 10.1016/0031-9422(90)85209-X

[B15] Itokawa H. Suto K. Takeya K. (1981). Studies on a novel P-coumaroyl glucoside of apigenin and on other flavonoids isolated from patchouli. *Chem. Pharm. Bull.* 29 254–256.

[B16] Jung U. J. Cho Y. Y. Choi M. S. (2016). Apigenin ameliorates dyslipidemia, hepatic steatosis and insulin resistance by modulating metabolic and transcriptional profiles in the liver of high fat diet-induced obese mice. *Nutrients* 8 305–321. 10.3390/nu8050305 27213439 PMC4882717

[B17] Kahn S. E. Hull R. L. Utzschneider K. M. (2006). Mechanisms linking obesity to insulin resistance and type 2 diabetes. *Nature* 444 840–846. 10.1038/nature05482 17167471

[B18] Karimi G. Sabran M. R. Jamaluddin R. Parvaneh K. Mohtarrudin N. Ahmad Z. (2015). The anti-obesity effects of *Lactobacillus casei* strain shirota versus orlistat on high fat diet-induced obese rats. *Food Nutr. Res.* 59:29273. 10.3402/fnr.v59.29273 26699936 PMC4689799

[B19] Kudo M. Sugawara A. Uruno A. Takeuchi K. Ito S. (2004). Transcription suppression of peroxisome proliferator-activated receptor 2 gene expression by tumor necrosis factor alpha via an inhibition of CCAAT/ enhancer-binding protein delta during the early stage of adipocyte differentiation. *Endocrinology* 145 4948–4956. 10.1210/en.2004-0180 15284209

[B20] Lakshmi B. S. Sujatha S. Anand S. Sangeetha K. N. Narayanan R. B. Katiyar C. (2009). Cinnamic acid, from the bark of *Cinnamomum cassia*, regulates glucose transport via activation of GLUT4 on L6 myotubes in a phosphatidylinositol 3-kinase-independent manner. *J. Diabetes* 1 99–106. 10.1111/j.1753-0407.2009.00022.x 20929506

[B21] Lampson M. A. Racz A. Cushman S. W. McGraw T. E. (2000). Demonstration of insulin-responsive trafficking of glut4 and vptr in fibroblasts. *J. Cell Sci.* 113 4065–4076. 11058093 10.1242/jcs.113.22.4065

[B22] Lehrke M. Lazar M. A. (2005). The many faces of PPARgamma. *Cell* 123 993–999. 10.1016/j.cell.2005.11.026 16360030

[B23] Leto D. Saltiel A. R. (2012). Regulation of glucose transport by insulin: traffic control of GLUT4. *Nat. Rev. Mol. Cell Biol.* 13 383–396. 10.1038/nrm3351 22617471

[B24] Li W. Yuan G. Pan Y. Wang C. Chen H. (2017). Network pharmacology studies on the bioactive compounds and action mechanisms of natural products for the treatment of diabetes mellitus: a review. *Front. Pharmacol.* 8:74. 10.3389/fphar.2017.00074 28280467 PMC5322182

[B25] Matsuo K. Ito M. Honda G. Qui T. Kiuchi F. (2003). Trypanocidal flavonoids from *Sophora flavescens*. *Nat. Med.* 57 253–255.

[B26] Memon R. A. Tecott L. H. Nonogaki K. Beigneux A. Moser A. H. Grunfeld C. (2000). Up-regulation of peroxisome proliferator-activated receptors (PPAR-α) and PPAR γ messenger ribonucleic acid expression in the liver in murine obesity: troglitazone induces expression of PPAR γ responsive adipose tissue-specific genes in the liver of obese diabetic mice. *Endocrinology* 141 4021–4031. 10.1210/endo.141.11.7771 11089532

[B27] Morán-Salvador E. López-Parra M. García-Alonso V. Titos E. Martínez-Clemente M. González-Périz A. (2011). Role for PPAR γ in obesity-induced hepatic steatosis as determined by hepatocyte- and macrophage-specific conditional knockouts. *FASEB J.* 25 2538–2550. 10.1096/fj.10-173716 21507897

[B28] Morgan B. J. Chai S. Y. Albiston A. L. (2011). GLUT4 associated proteins as therapeutic targets for diabetes. *Recent Pat. Endocr. Metab. Immune Drug Discov.* 5 25–32. 10.2174/187221411794351914 22074575

[B29] Moroki T. Yoshikawa Y. Yoshizawa K. Tsubura A. Yasui H. (2013). Morphological characterization of systemic changes in KK-Ay mice as an animal model of type 2 diabetes. *In Vivo* 27 465–472. 23812216

[B30] Nolan C. J. Damm P. Prentki M. (2011). Type 2 diabetes across generations: from pathophysiology to prevention and management. *Lancet* 378 169–181. 10.1016/S0140-6736(11)60614-4 21705072

[B31] Nyenwe E. A. Jerkins T. W. Umpierrez G. E. Kitabchi A. E. (2011). Management of type 2 diabetes: evolving strategies for the treatment of patients with type 2 diabetes. *Metabolism* 60 1–23. 10.1016/j.metabol.2010.09.010 21134520 PMC3746516

[B32] Park C. J. Lee H. A. Han J. S. (2016). Jicama (*Pachyrhizus erosus*) extract increases insulin sensitivity and regulates hepatic glucose in C57BL/Ksj-db/db mice. *Clin. Biochem. Nutr.* 58 56–63. 10.3164/jcbn.15-59 26798198 PMC4706093

[B33] Peng T. Zhao F. R. Chen X. Y. Jiang G. H. Wang S. N. (2016). Chemical study of the Chinese medicine Pi Han Yao. *Biomed. Rep.* 4 219–222. 10.3892/br.2016.566 26893842 PMC4734221

[B34] Ramachandran V. Saravanan R. (2015). Glucose uptake through translocation and activation of GLUT4 in PI3K/Akt signaling pathway by asiatic acid in diabetic rats. *Hum. Exp. Toxicol.* 34 884–893. 10.1177/0960327114561663 26286522

[B35] Rauter A. P. Martins A. Borges C. Mota-Filipe H. Pinto R. Sepodes B. (2010). Antihyperglycemic and protective effects of flavonoids on streptozotocin-induced diabetic rats. *Phytother. Res.* 24 S133–S138. 10.1002/ptr.3017 20309949

[B36] Ruderman N. B. Saha A. K. Vavvas D. Witters L. A. (1999). Malonyl-CoA, fuel sensing, and insulin resistance. *Am. J. Physiol.* 276 E1–E18. 9886945 10.1152/ajpendo.1999.276.1.E1

[B37] Saltiel A. R. Kahn C. R. (2001). Insulin signalling and the regulation of glucose and lipid metabolism. *Nature* 414 799–806. 10.1038/414799a 11742412

[B38] Shen N. Huan Y. Shen Z. F. (2012). Berberine inhibits mouse insulin gene promoter through activation of AMP activated protein kinase and may exert beneficial effect on pancreatic β-cell. *Eur. J. Pharmacol.* 694 120–126. 10.1016/j.ejphar.2012.07.052 22955013

[B39] Srinivasan K. Ramarao P. (2007). Animal models in type 2 diabetes research: an overview. *Indian J. Med. Res.* 136 451–472. 17496368

[B40] Tai Z. Cai L. Dai L. Dong L. H. Wang M. F. Yang Y. B. (2011). Antioxidant activity and chemical constituents of edible flower of *Sophora viciifolia*. *Food Chem.* 126 1648–1654. 10.1016/j.foodchem.2010.12.048 25213940

[B41] Thong F. S. Dugani C. B. Klip A. (2005). Turning signals on and off: glut4 traffic in the insulin-signaling highway. *Physiology (Bethesda)* 20 271–284. 10.1152/physiol.00017.2005 16024515

[B42] Tsuchiya A. Kanno T. Nishizaki T. (2013). PKC𝜀-dependent and PKCλ/ι and -ζ-independent manner. *Life Sci.* 93 240–246. 10.1016/j.lfs.2013.06.014 23800645

[B43] Xiong M. R. Huang Y. Liu Y. J. Huang M. Song G. J. Ming Q. (2018). Antidiabetic activity of ergosterol from *Pleurotus ostreatus* in KK-Ay mice with spontaneous type 2 diabetes mellitus. *Mol. Nutr. Food Res.* 62:1700444. 10.1002/mnfr.201700444 29080247

[B44] Yang J. Wang C. Lin Q. X. Mei Z. N. Yang G. Z. Yang X. Z. (2014). Lavandulyl flavonoids with sodium-dependent glucose cotransporter 2 inhibitory activity from Sophora flavescens. *J. Huazhong Norm. Univ.* 48 520–524. 10.19603/j.cnki.1000-1190.2014.04.013

[B45] Yang X. Z. Huang M. Yang J. Wang J. L. Zheng S. J. Ma X. H. (2017). Activity of isoliensinine in improving the symptoms of type 2 diabetic mice via activation of AMP-activated kinase and regulation of PPARγ. *J. Agric. Food Chem.* 65 7168–7178. 10.1021/acs.jafc.7b01964 28745497

[B46] Yu S. Matsusue K. Kashireddy P. Cao W. Q. Yeldandi V. Yeldandi A. V. (2003). Adipocyte-specific gene expression and adipogenic steatosis in the mouse liver due to peroxisome proliferator-activated receptor gamma1 (PPAR gamma1) overexpression. *J. Biol. Chem.* 278 498–505. 10.1074/jbc.M210062200 12401792

[B47] Zhang B. B. Zhou G. Li C. (2009). AMPK: an emerging drug target for diabetes and the metabolic syndrome. *Cell Metab.* 9 407–416. 10.1016/j.cmet.2009.03.012 19416711

[B48] Zhao P. Yang L. Lopez J. A. Fan J. Burchfield J. G. Bai L. (2009). Variations in the requirement for v-SNAREs in GLUT4 trafficking in adipocytes. *J. Cell Sci.* 122 3472–3480. 10.1242/jcs.047449 19759285

[B49] Zhao R. N. (2004). *Chinese Herbal Medicine Resources In Gansu Province.* Lanzhou: Gansu Science and Technology Press, 773–775.

[B50] Zheng S. J. Deng S. H. Huang Y. Huang M. Zhao P. Ma X. H. (2017). Anti-diabetic activity of a polyphenol-rich extract from Phellinus igniarius in KK-Ay mice with spontaneous type 2 diabetes mellitus. *Food Funct.* 9 614–623. 10.1039/c7fo01460k 29271444

[B51] Zisman A. Peroni O. D. Abel E. D. Michael M. D. Mauvais-Jarvis F. Lowell B. B. (2000). Targeted disruption of the glucose transporter 4 selectively in muscle causes insulin resistance and glucose intolerance. *Nat. Med.* 6 924–928. 10.1038/78693 10932232

