# Supplementary material for: Antidiabetic Activity of a Flavonoid-Rich Extract From Sophora davidii (Franch.) Skeels in KK-Ay Mice via Activation of AMP-Activated Protein Kinase
Source: Front Pharmacol. 2018 Jul 16;9:760. doi: 10.3389/fphar.2018.00760 (PMC6055046; doi:10.3389/fphar.2018.00760)
Supplement: Supplementary file 1 [file Presentation_1.pdf]

Antidiabetic activity of a flavonoid-rich extract from *Sophora davidi* (Franch.) Skeels in KK-Ay Mice via activation of AMP-activated protein kinase

Yun Huang[1], Ji Hao[1], Di Tian[2], Yanzhang Wen[1], Ping Zhao[2], Hao Chen[3], Yibin Lv[1], Xinzhou Yang[1,*]

[1]School of Pharmaceutical Sciences, South-Central University for Nationalities, Wuhan 430074, China

[2]School of Life Sciences, South-Central University for Nationalities, 182 Min-Zu Road, Wuhan 430074, China

[3]College of Pharmacy, Guangxi University of Chinese Medicine, Nanning 530001, China

[*]Corresponding authors at: School of Pharmaceutical Sciences, South-Central University for Nationalities, 182 Minzu Road, Wuhan 430074, China, Tel.: +86 27 67841196, Fax: +86 27 67841196.

E-mail addresses: xzyang@mail.scuec.edu.cn (X.Z. Yang).

Supporting Information Contents:

S1. Screening methodology validation

S2. Changes of FBG levels, body weight and food intake of KK-Ay mice during 4 weeks of treatment

S3. ^{1}H-NMR spectrum of Compound 1

S4. ^{13}C-NMR spectrum of Compound 1

S5. ESIMS spectrum of Compound 1 (Positive and negative mode)

S6. ^{1}H-NMR spectrum of Compound 2

S7. ^{13}C-NMR spectrum of Compound 2

S8. ESIMS spectrum of Compound 2 (Positive and negative mode)

S9. ^{1}H-NMR spectrum of Compound 3

S10. ^{13}C-NMR spectrum of Compound 3

S11. ESIMS spectrum of Compound 3 (Positive and negative mode)

S12. ^{1}H-NMR spectrum of Compound 4

S13. ^{13}C-NMR spectrum of Compound 4

S14. ESIMS spectrum of Compound 4 (Positive and negative mode)

S1. Screening methodology validation

There have several papers reported that IRAP-mOrange and GLUT4-eGFP could be applied to detect the GLUT4 translocation in L6 (Wang et al., 2009; Zhou et al., 2016; Huang et al., 2016) and 3T3-L1 cells (Bai et al., 2007; Jiang et al. 2008). In order to validate the feasibility of our IRAP translocation assay for discovering potential hypoglycemic agents, we have observed the effects when the GLUT4-eGFP or IRAP-marked L6 cells treated with insulin and berberine which are definitely pharmacodynamic GLUT4 agonists. L6 cells which stably express IRAP-mOrange and GLUT4-eGFP were cultured in MEM-α supplemented with 10% fetal bovine serum and 1% antibiotics (100 U/mL penicillin and 100 μg/mL streptomycin) at 37 oC in 5% CO_2. L6 cells was seeded in 48 well plates, and incubated until 100% confluence and then starved in serum-free α-MEM for 2 h. Afterwards, L6 cells were treated with insulin (10 nM) and berberine (5 μM). The cells were taken photos with a laser-scanning confocal microscope LSM 510 (Carl Zeiss, Jena, Germany) to supervise the IRAP-mOrange and GLUT4-eGFP translocation. And the images were captured with 555 nm excitation laser every 10 seconds in first 5 minutes and then every 5 minutes in later 30 minutes. During the experiment, as time went on, we could observe the green and red fluorescence enhanced significantly after treating with insulin and berberine in L6 cells (Fig. 1). The results showed that GLUT4 and IRAP simultaneously translocated onto the plasma membrane in 30 min when adding the GLUT4 agonist. GLUT4 has mainly been recruited to the PM throughout to the GLUTs storage vesicles (GSV). Three main proteins stored in GSV are GLUT4, IRAP, and Sortilin (Shi et al., 2005). It was reported that IRAP and GLUT4 displayed a strong colocalization (Kumar et al., 2010; Rubin et al., 2009) in many researches. Thus, detecting the IRAP can indirectly reflect the situation of GLUT4. So our results could be explained that detecting the IRAP-mOrange fluorescence could indirectly reflect the GLUT4 translocation. As the red fluorescence is more conspicuous than green fluorescence for observation, so we choose the IRAP-mOrange fluorescence assay for reflecting GLUT4 translocation.

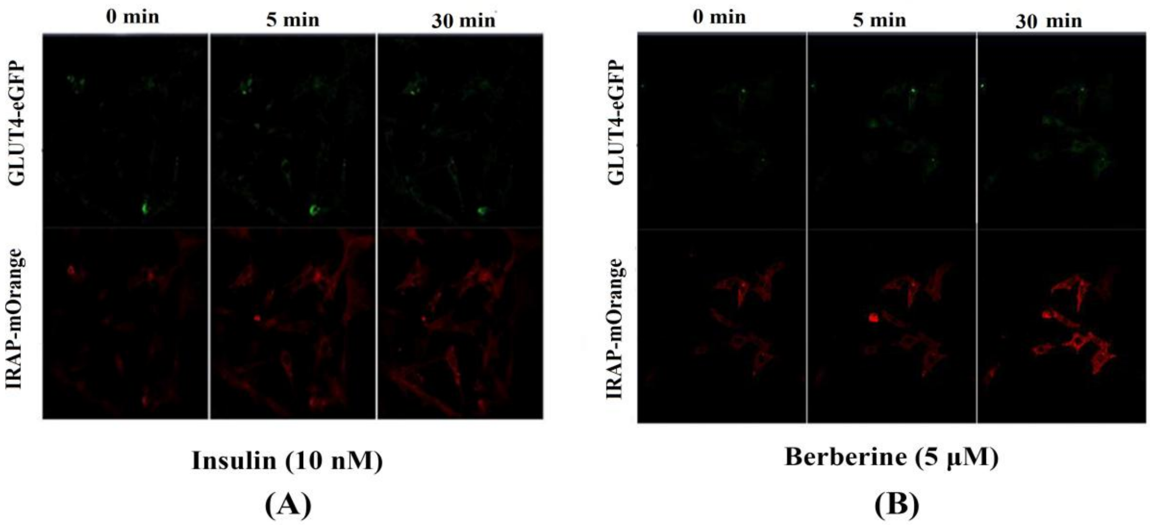

Figure S1 L6 cells were infected with IRAP-mOrange and GLUT4-eGFP in order to detect

externalized GLUT4 translocation by confocal microscopy. (A) Confocal images in L6 cells incubated in the absence (0 min) or presence of insulin for 5min, 30 minutes. (B) Confocal images in L6 cells incubated in the absence (0 min) or presence of berberine for 5 min, 30 minutes.

References

Bai, L., Wang, Y., Fan, J., Chen, Y., Ji, W., Qu, A., et al. (2007). Dissecting multiple steps of GLUT4 trafficking and identifying the sites of insulin action. *Cell. Metab*. 5, 47-57. doi: 10.1016/j.cmet.2006.11.013

Huang, M., Zhao, P., Xiong, M. R., Zhou, Q., Zheng, S. J., Ma , X. H., et al. (2016). Antidiabetic activity of perylenequinonoid-rich extract from *Shiraia bambusicola* in KK-Ay Mice with Spontaneous Type 2 Diabetes Mellitus. *J. Ethnopharmac.* 191, 71-81. doi: 10.1016/j.jep.2016.06.018

Jiang, L., Fan, J., Bai, L., Wang, Y., Chen, Y., Yang, L., et al. (2008). Direct quantification of fusion rate reveals a distal role for AS160 in insulin-stimulated fusion of GLUT4 storage vesicles. *J. Biol. Chem.* 283, 8508-8516. doi: 10.1074/jbc.M708688200

Kumar, A., Lawrence, Jr., Jung, D. Y., Ko, H. J., Keller, S. R., Kim, J. K., et al. (2010). Fat cellspecific ablation of rictor in mice impairs insulin-regulated fat cell and whole-body glucose and lipidmetabolism. *Diabetes.* 59, 1397–1406. doi: 10.2337/db09-1061

Rubin, B. R., and Bogan, J. S. (2009). Intracellular retention and insulinstimulated mobilization of GLUT4 glucose transporters. *Vitam. Horm.* 80, 155–192. doi: 10.1016/S0083-6729(08)00607-9

Shi, J., and Kandror, K. V. (2005). Sortilin is essential and sufficient for the formation of glut4 storage vesicles in 3T3-L1 adipocytes. *Dev. Cell.* 9, 99–108. doi: 10.1016/j.devcel.2005.04.004

Wang, X., Qu, F., Chen, Z., Liang, T., and Qu, A. (2009). Labeling and imaging of GLUT4 in live L6 cells with quantum dots. *Biochem. Cell. Biol.* 87, 687-694. doi: 10.1139/o09-041

Zhou, Q., Yang, X. Z., Xiong, M. R., Xu, X. L., Zhen, L., Chen, W. W., et al. (2016). Chloroquine Increases Glucose Uptake via Enhancing GLUT4 Translocation and Fusion with the Plasma Membrane in L6 Cells. *Cell Physiol. Biochem.* 38, 2030-2040. doi: 10.1159/000445562

S2. Changes of FBG levels, body weight and food intake of KK-Ay mice during 4 weeks of treatment.

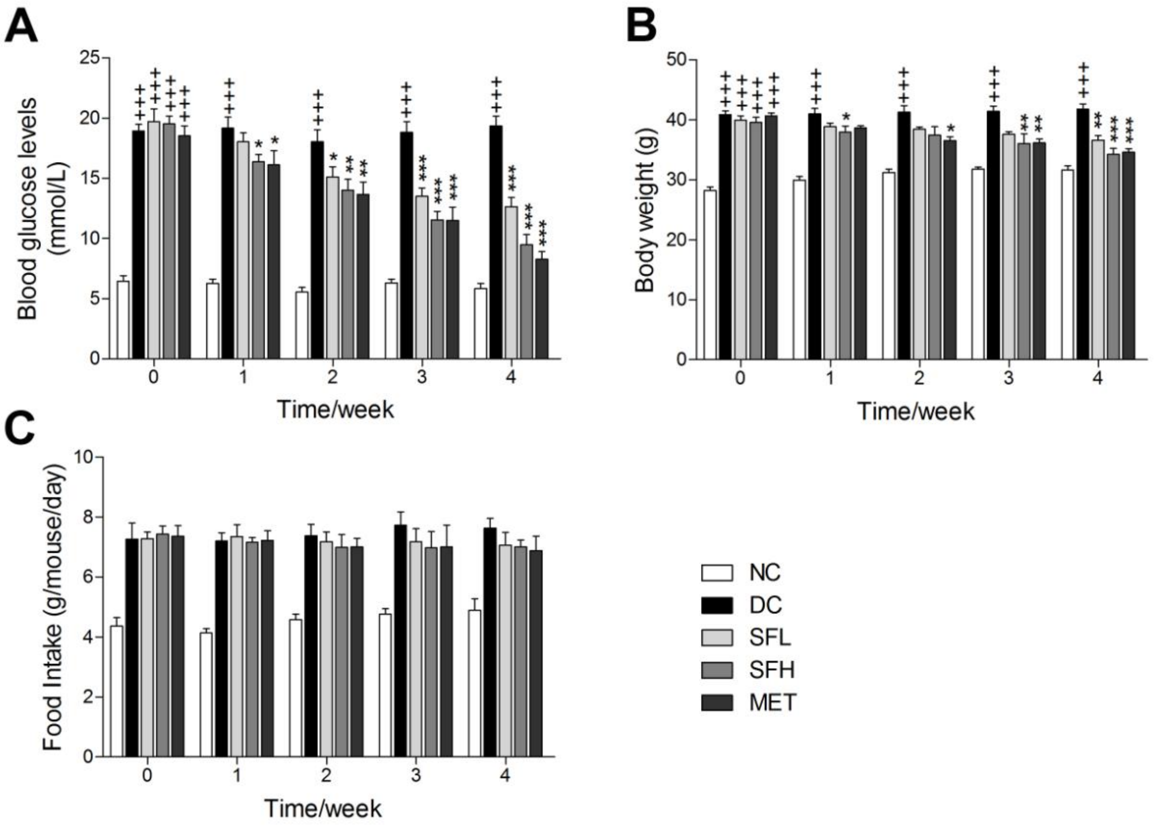

Figure S2 The dynamic changes of FBG levels (A), body weight (B) and food intake (C) of KK-Ay mice during 4 weeks of treatment with SD-FRE. Data are means ± SEM (n = 8). $^{+++}P < 0.001$ versus NC group, $^{*}P < 0.05$, $^{**}P < 0.01$, $^{***}P < 0.001$ versus DC group.

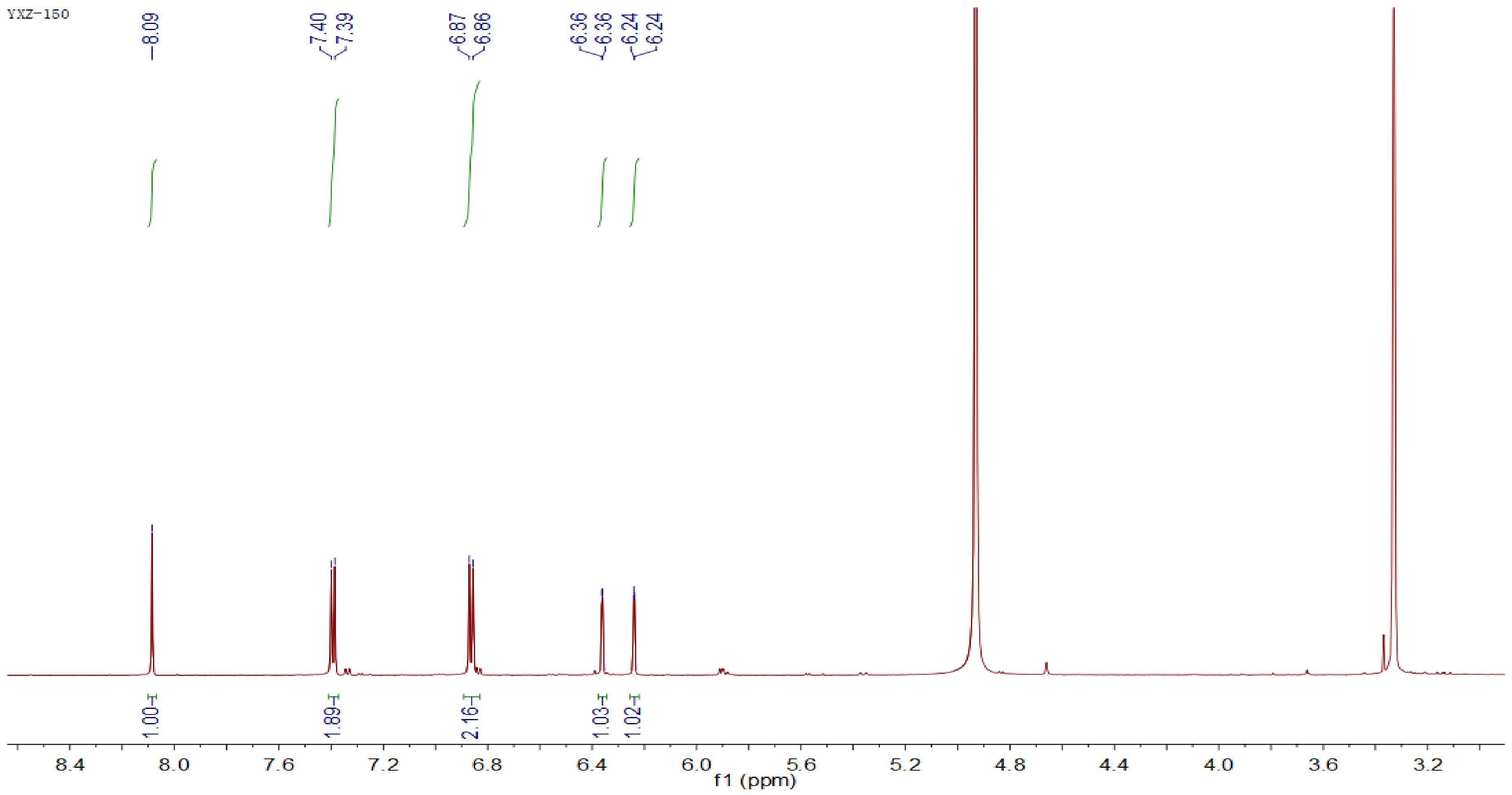

S3. ^{1}H-NMR spectrum of apigenin

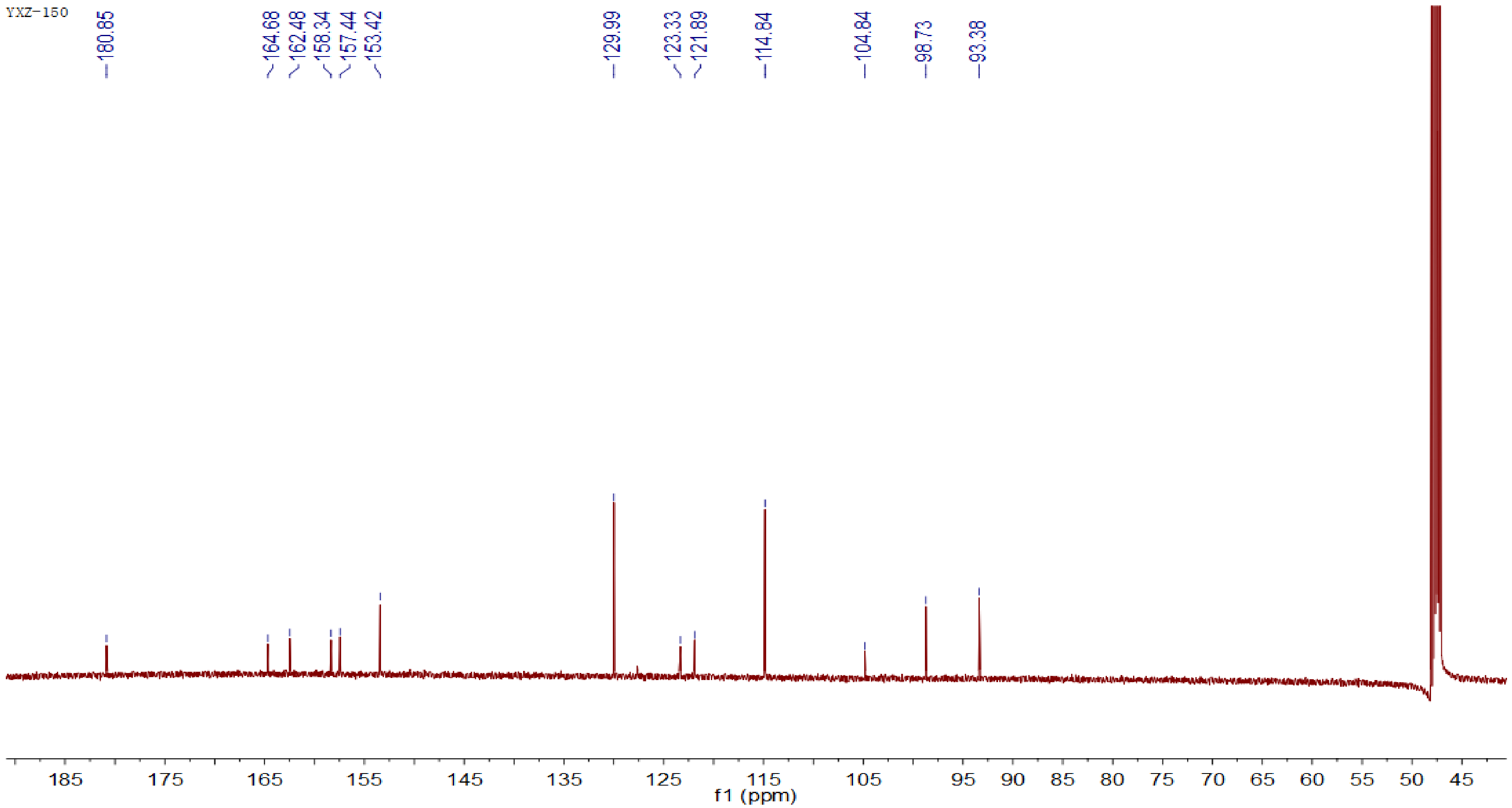

S4. ^{13}C-NMR spectrum of apigenin

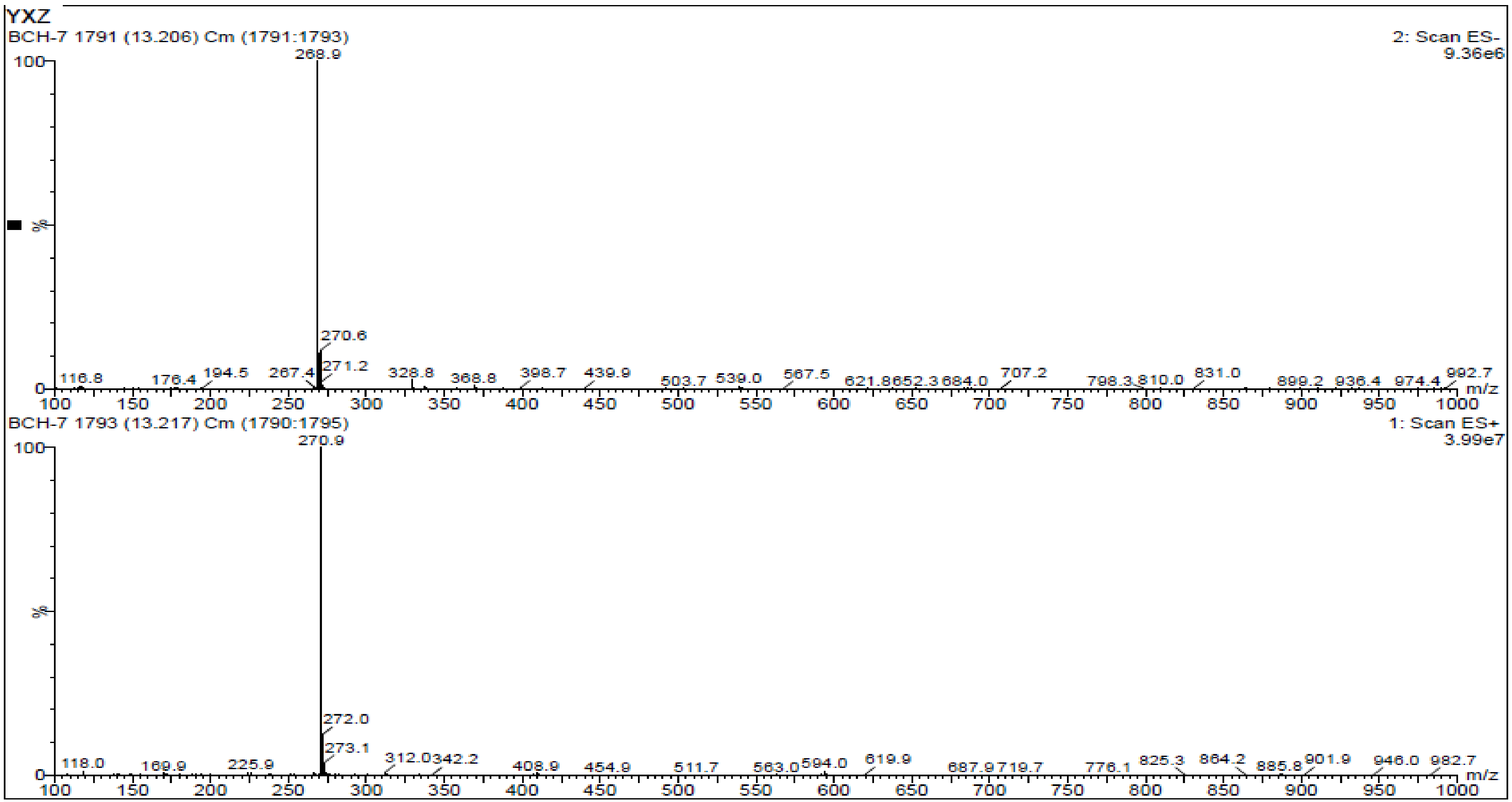

S5. ESIMS spectrum of apigenin (Positive and negative mode)

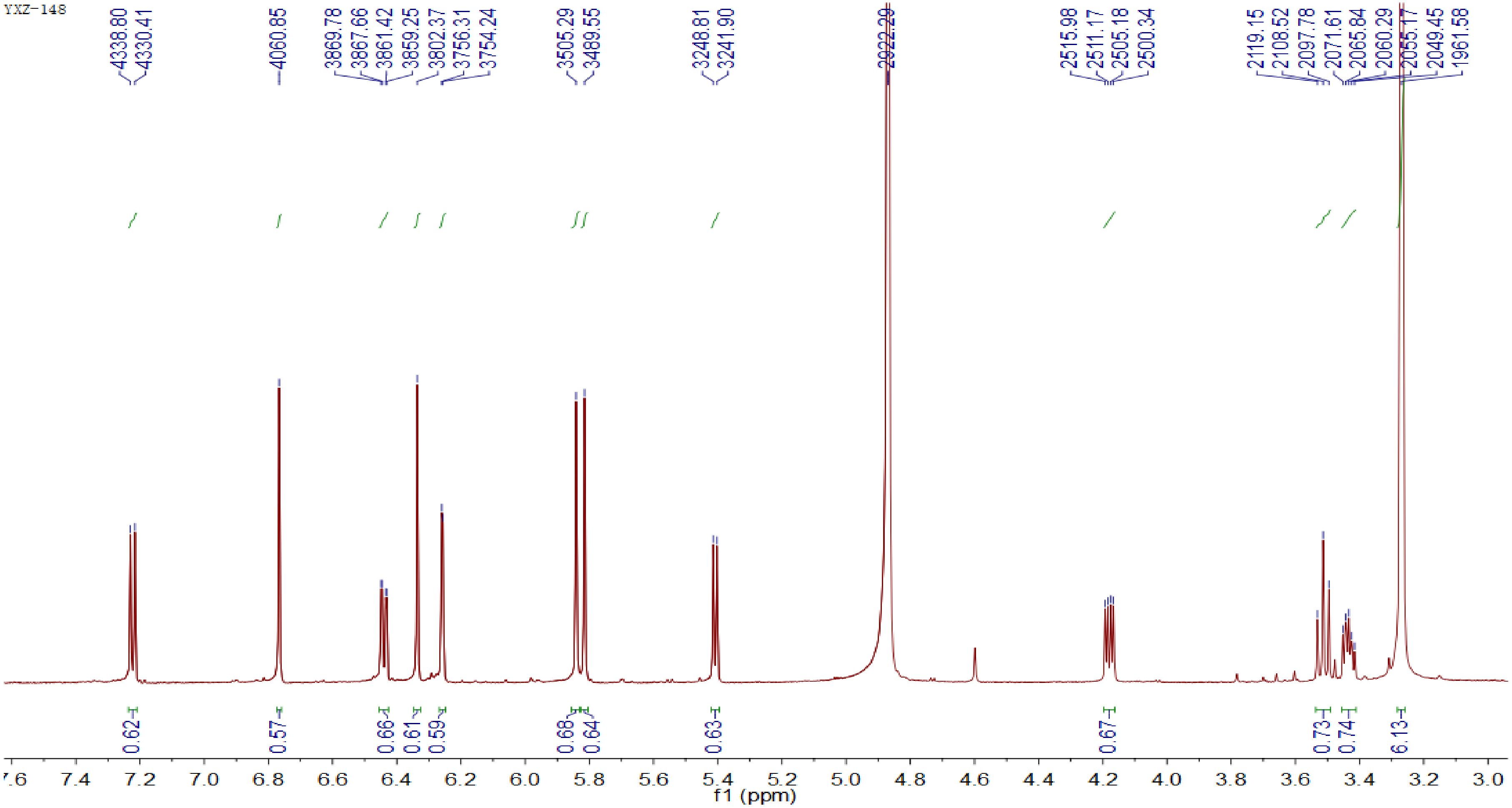

S6. ^{1}H-NMR spectrum of maackiain

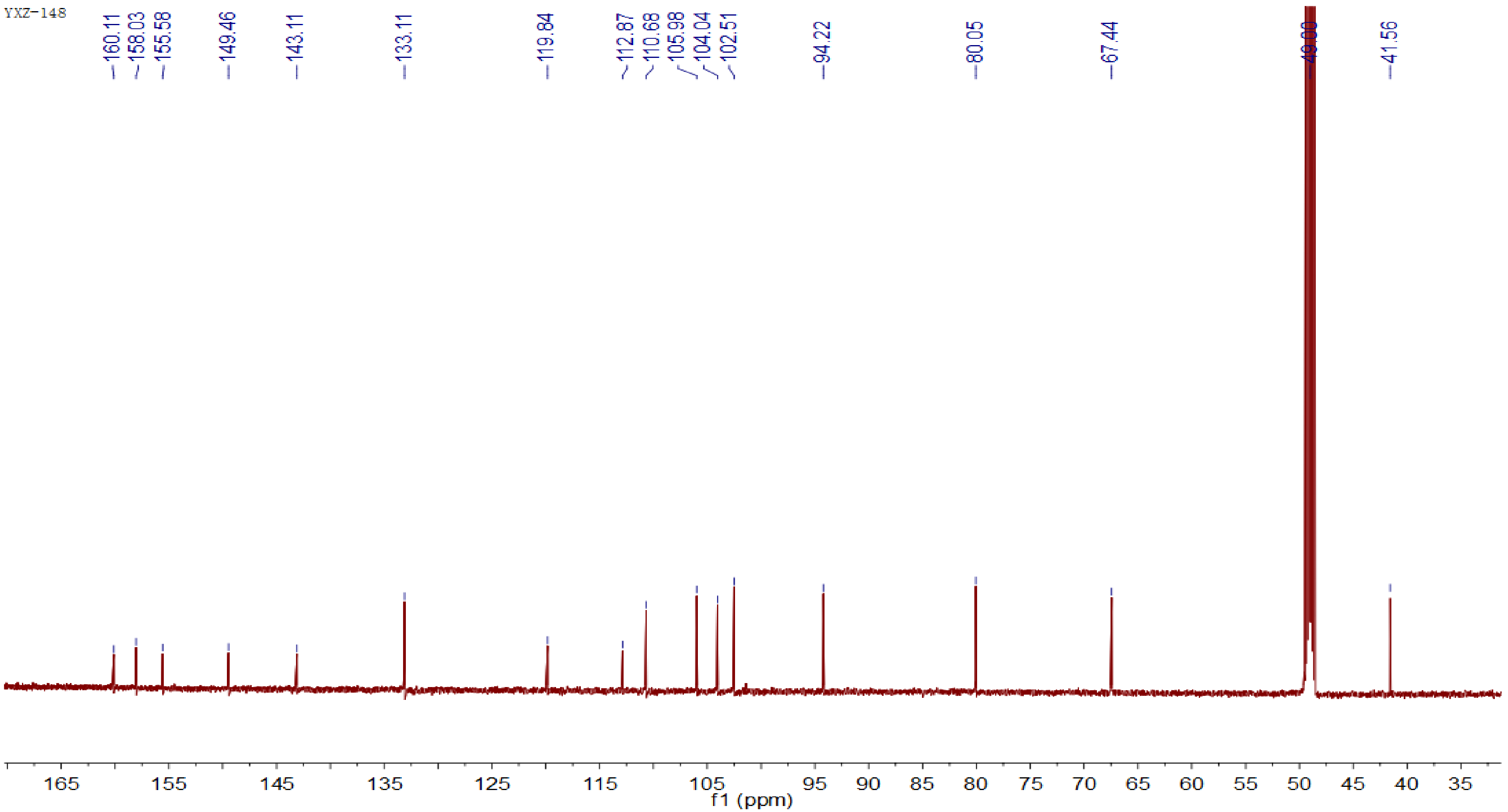

S7. ^{13}C-NMR spectrum of maackiain

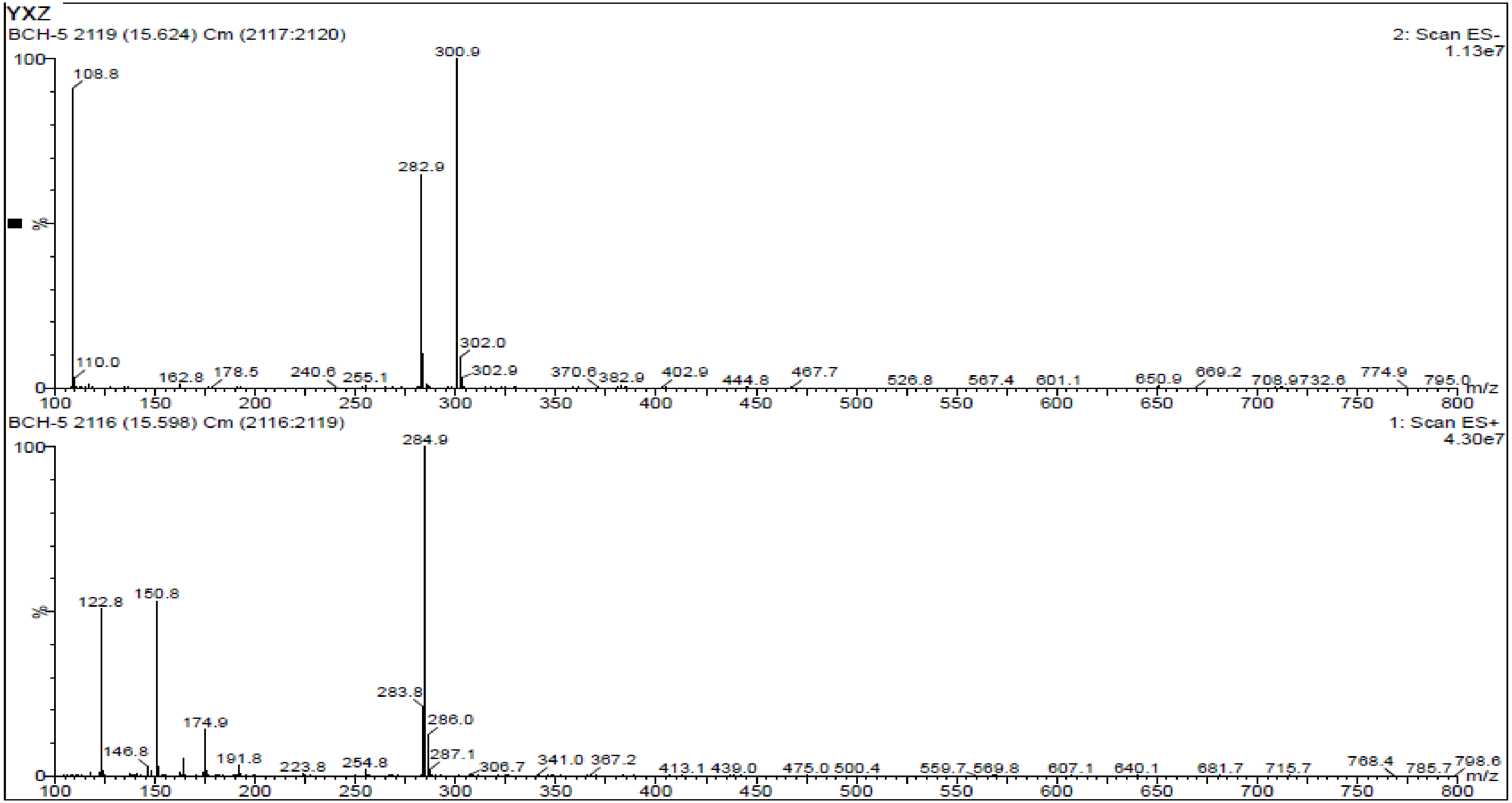

S8. ESIMS spectrum of maackiain (Positive and negative mode)

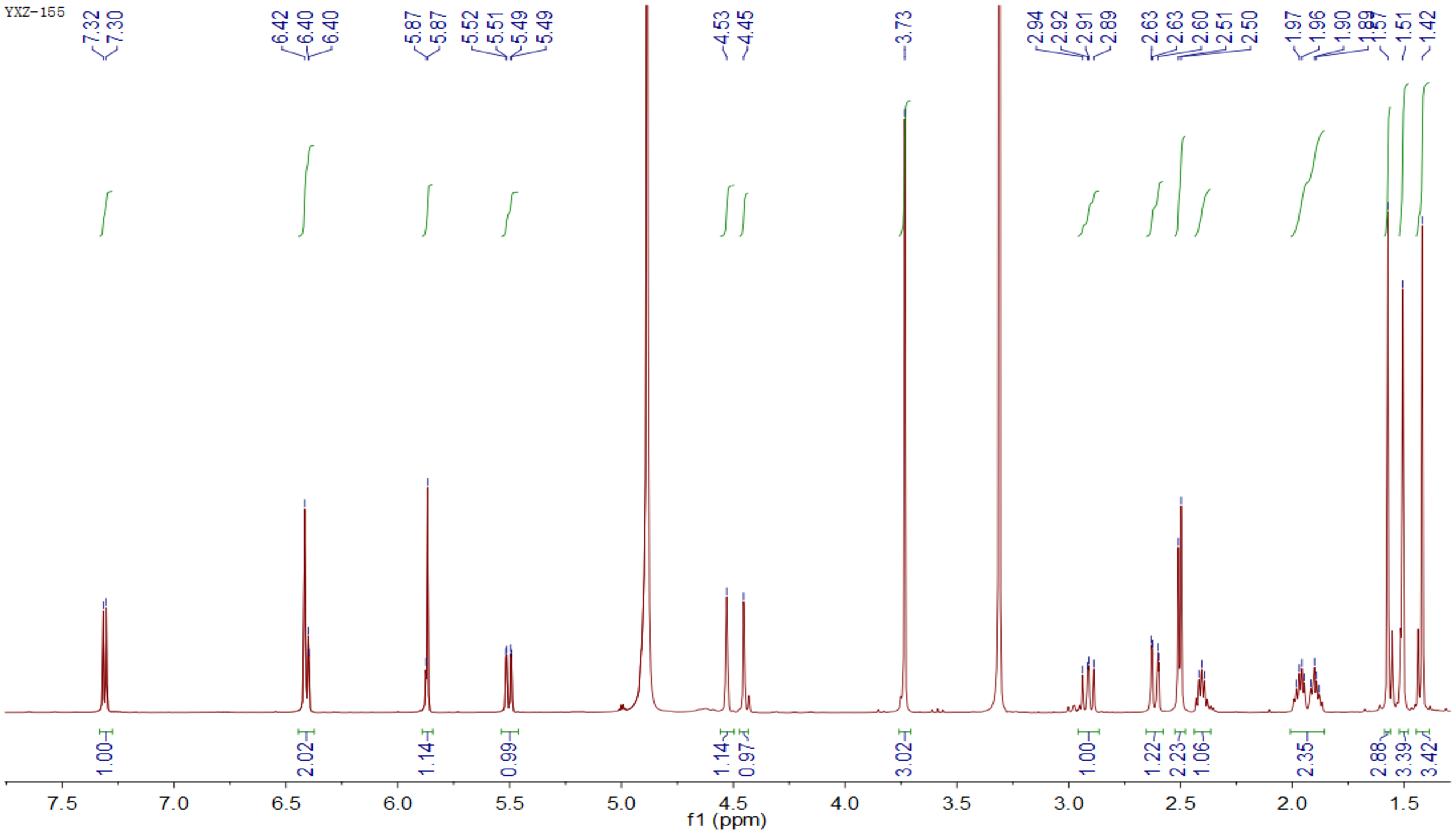

S9. ^{1}H-NMR spectrum of leachianone A

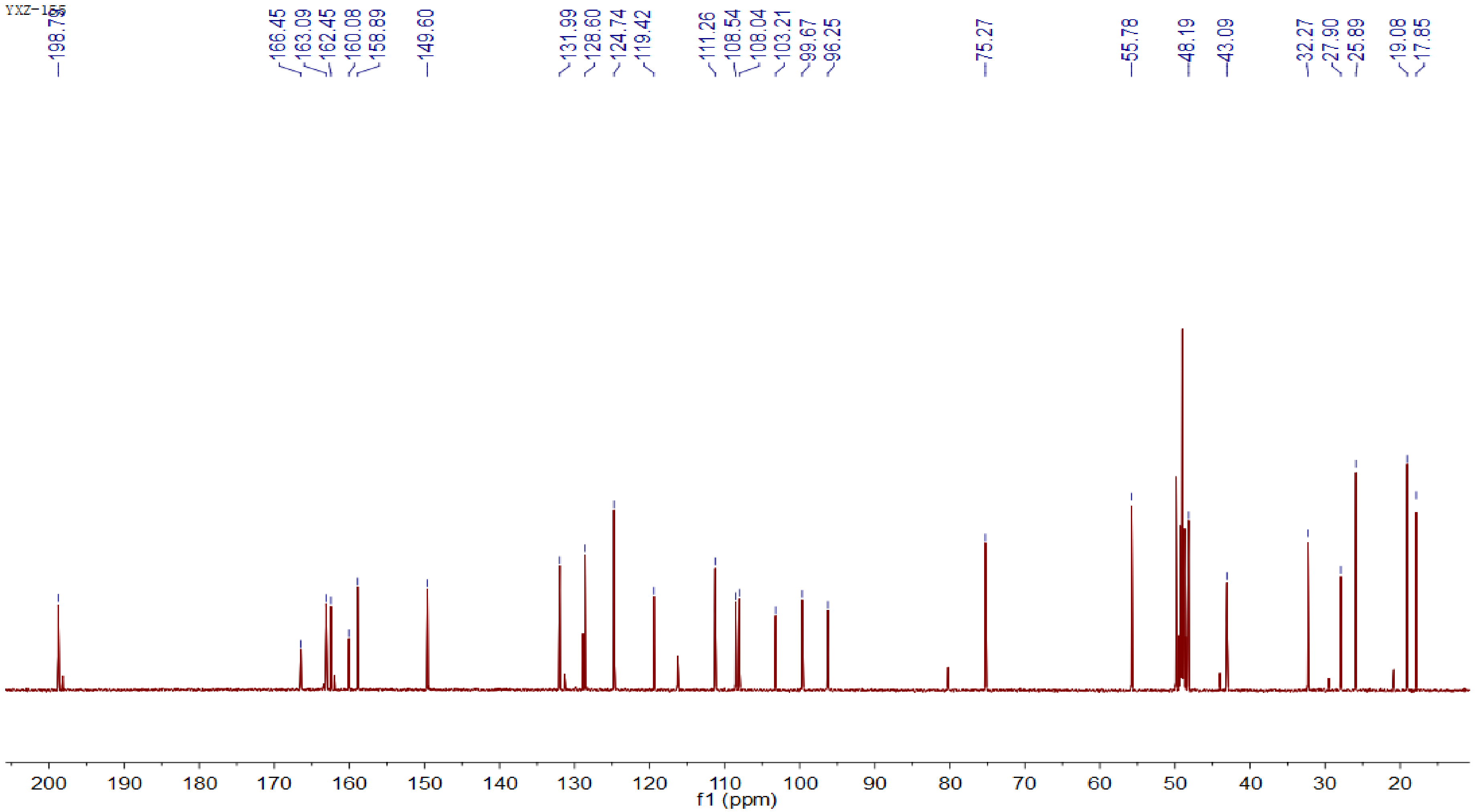

S10. ^{13}**C-NMR spectrum of leachianone A**

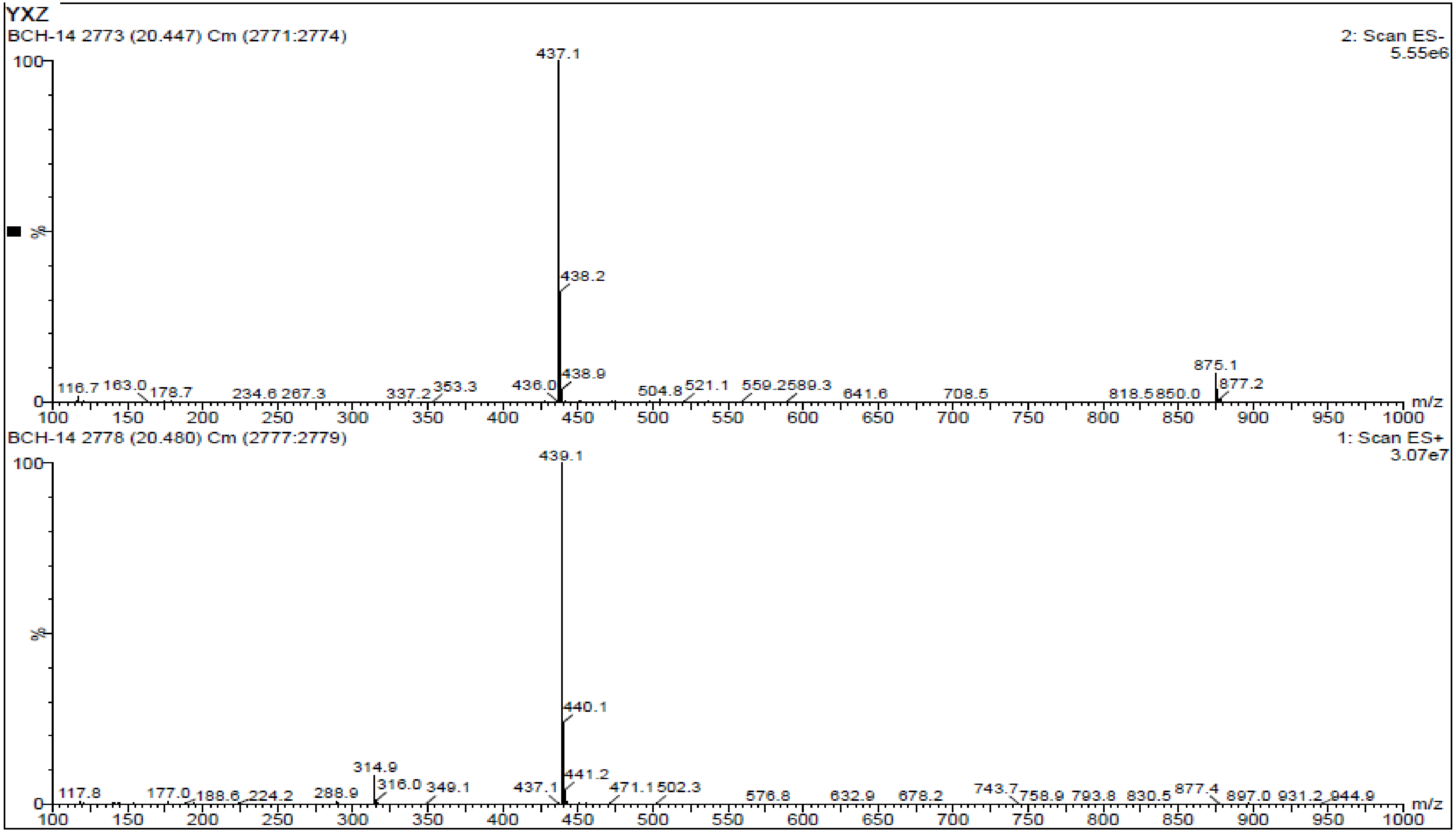

S11. ESIMS spectrum of leachianone A (Positive and negative mode)

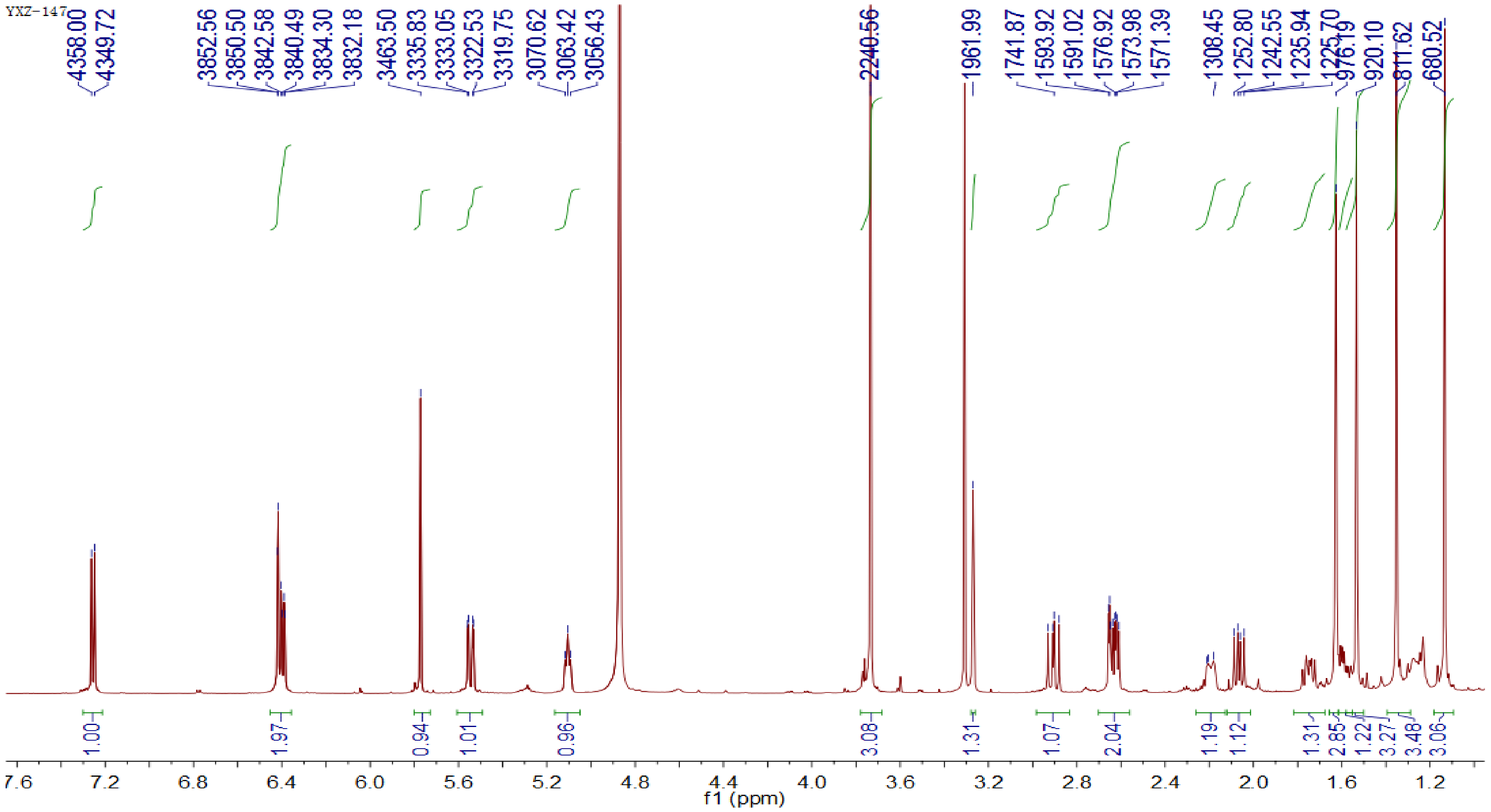

S12. ^{1}H-NMR spectrum of leachianone B

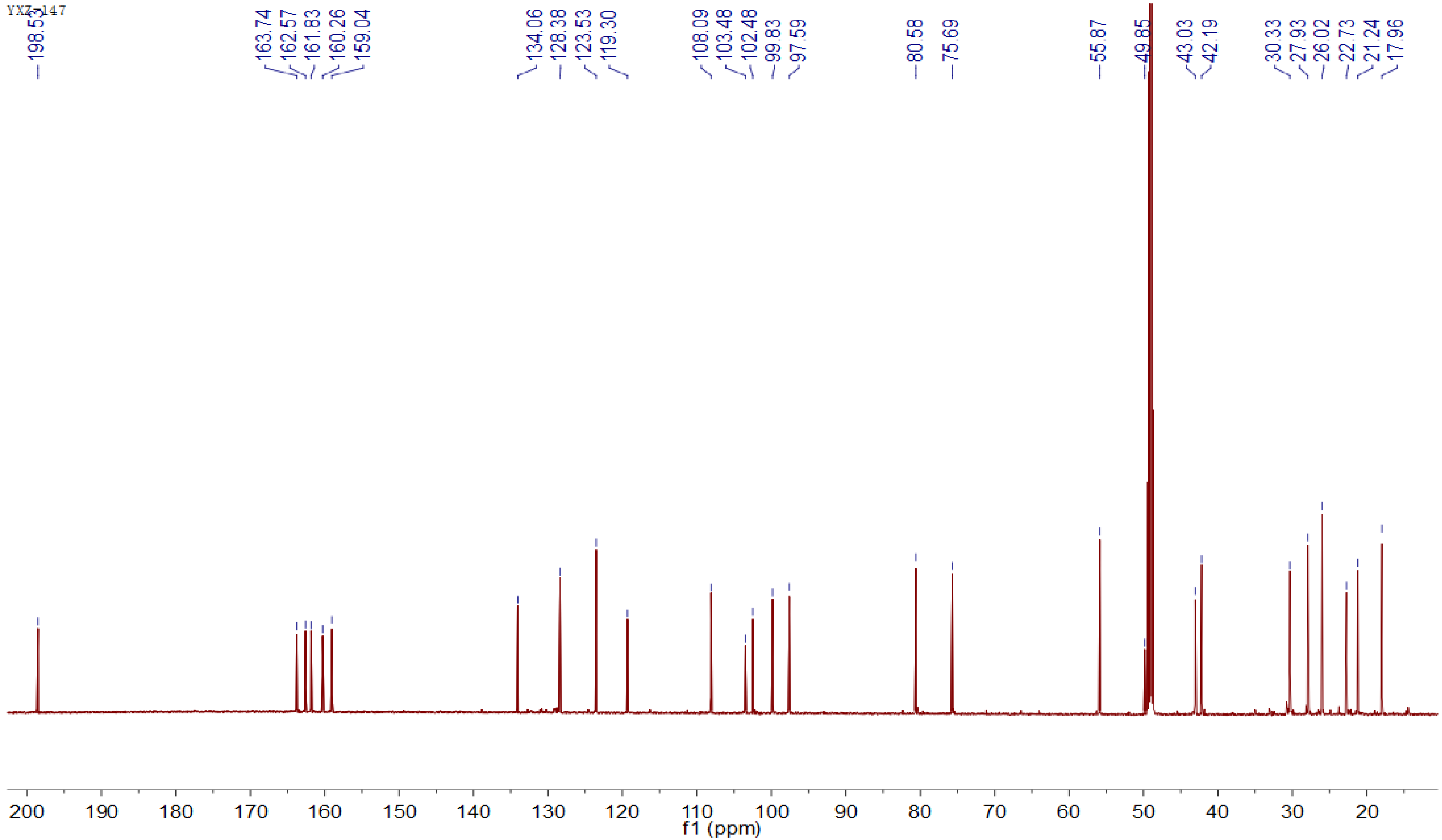

S13. ^{13}C-NMR spectrum of leachianone B

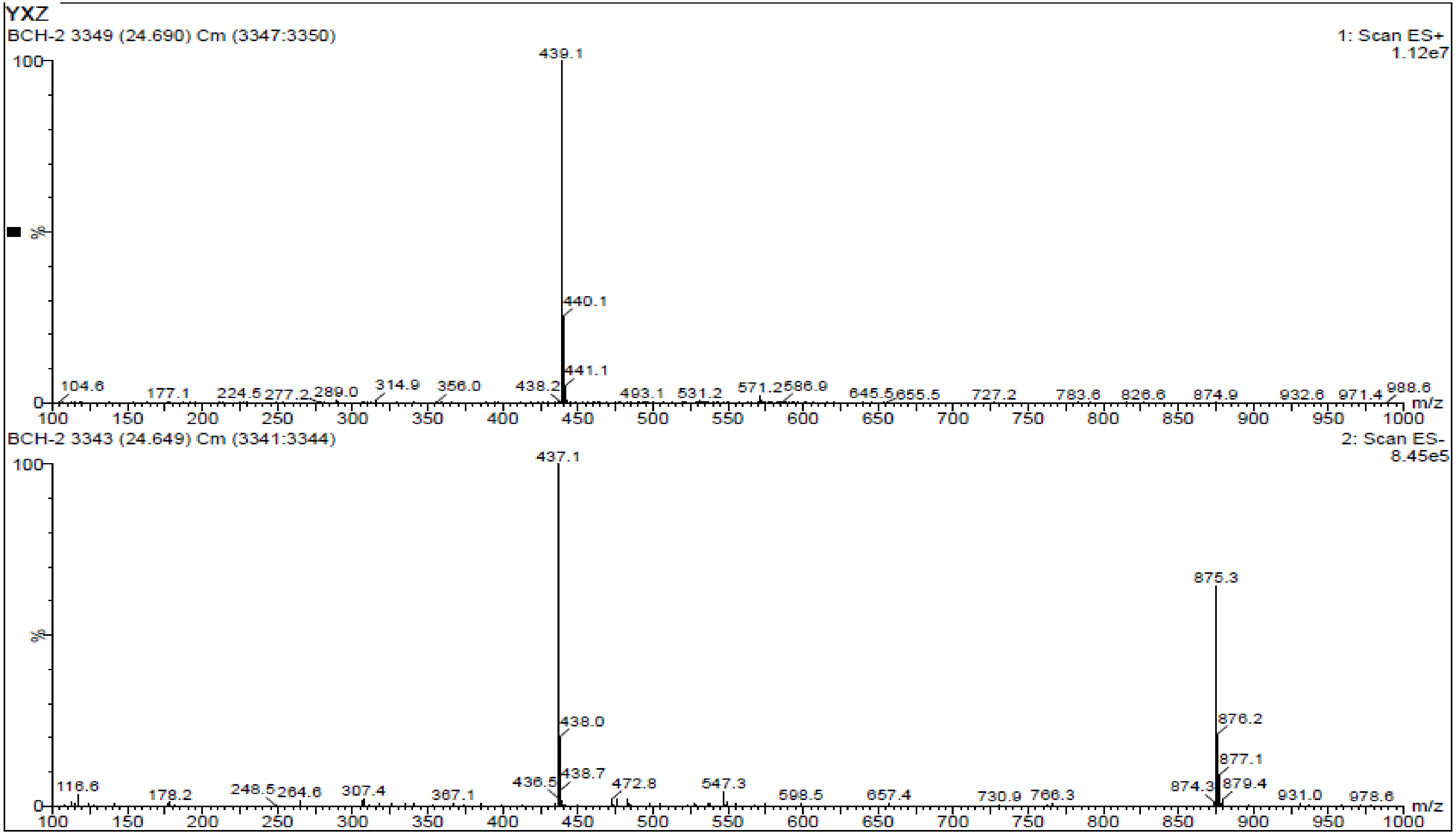

S14. ESIMS spectrum of leachianone B (Positive and negative mode)
